# Delivering Microrobots in the Musculoskeletal System

**DOI:** 10.1007/s40820-024-01464-8

**Published:** 2024-07-22

**Authors:** Mumin Cao, Renwang Sheng, Yimin Sun, Ying Cao, Hao Wang, Ming Zhang, Yunmeng Pu, Yucheng Gao, Yuanwei Zhang, Panpan Lu, Gaojun Teng, Qianqian Wang, Yunfeng Rui

**Affiliations:** 1 Department of Orthopaedics, Zhongda Hospital, School of Medicine, Southeast University, Nanjing, Jiangsu People’s Republic of China; 2 School of Medicine, Southeast University, Nanjing, 210009 People’s Republic of China; 3 Orthopaedic Trauma Institute (OTI), Southeast University, Nanjing, 210009 People’s Republic of China; 4 Jiangsu Key Laboratory for Design and Manufacture of Micro-Nano Biomedical Instruments, School of Mechanical Engineering, Southeast University, Nanjing, 210009 People’s Republic of China; 5 Center of Interventional Radiology and Vascular Surgery, Department of Radiology, Zhongda Hospital, School of Medicine, Southeast University, Nanjing, 210009 People’s Republic of China

**Keywords:** Microrobot, Musculoskeletal system, Targeted delivery, Microrobotic systems, Magnetic actuation

## Abstract

Disorders of the musculoskeletal system are the major contributors to the global burden of disease and current treatments show limited efficacy. Patients often suffer chronic pain and might eventually have to undergo end-stage surgery. Therefore, future treatments should focus on early detection and intervention of regional lesions. Microrobots have been gradually used in organisms due to their advantages of intelligent, precise and minimally invasive targeted delivery. Through the combination of control and imaging systems, microrobots with good biosafety can be delivered to the desired area for treatment. In the musculoskeletal system, microrobots are mainly utilized to transport stem cells/drugs or to remove hazardous substances from the body. Compared to traditional biomaterial and tissue engineering strategies, active motion improves the efficiency and penetration of local targeting of cells/drugs. This review discusses the frontier applications of microrobotic systems in different tissues of the musculoskeletal system. We summarize the challenges and barriers that hinder clinical translation by evaluating the characteristics of different microrobots and finally point out the future direction of microrobots in the musculoskeletal system.

## Highlights

- A systematic review of recent advances of microrobots applied in the musculoskeletal system with an emphasis on design strategies of microrobotic systems for tissue regeneration.
- The fabrication, motion and control, and image-guided delivery of microrobots in the musculoskeletal system are reviewed based on the up-to-date works.
- Prospects and challenges for future clinical translation of microrobots in the musculoskeletal system and regenerative medicine are discussed.

## Graphical abstract

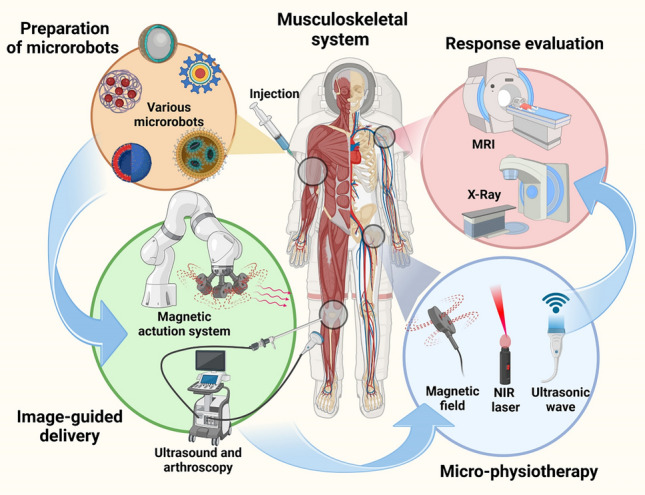

## Introduction

The musculoskeletal system consists of bones, muscles, cartilages, tendons, ligaments, and other connective tissues that hold other organs together, providing form, support, stability, and movement for the body [1]. Musculoskeletal system disorders (MSDs) include sarcopenia, fractures, osteoporosis, osteoarthritis (OA), tendon/ligament injuries, and various acute or chronic anatomical disorders, which are characterized by loss of muscle mass and strength, increased bone fragility, decreased cartilage resilience, and reduced tendon/ligament elasticity [2]. As a group of diseases that are common in people of all ages and social classes, these diseases are often associated with low life quality, disability, and even death [3]. In 2019, more than 1.6 billion adults aged 15–64 years suffered from a disease requiring rehabilitation, with MSDs accounting for approximately two-thirds of them [4]. In the context of an aging global population, the social and individual burden of MSDs is also increasing every day [1, 5]. Conventional medications like nonsteroidal anti-inflammatory drugs (NSAID) provide only symptomatic relief and limited tissue repair in treating MSDs, while increasing the risk of cardiovascular diseases and osteonecrosis [6–8]. With the development of tissue engineering and regenerative medicine, more and more technologies have emerged for therapeutic use, including stem cells [9, 10], biomaterials [11, 12], exosomes (EXOs) [13, 14], gene therapy [15–17], and many others. For instance, as a tissue that lacks blood vessels and nerves, articular cartilage is difficult to self-heal after injury and conservative treatments like NSAID and physiotherapy presents poor therapeutic effects [18]. In the last 20 years, various tissue engineering technologies like matrix-induced autologous chondrocyte implantation (MACI), which involves the combination of scaffolds, cells, and/or bioactive factors, were developed to effectively treat cartilage defects in clinic [19, 20]. Compared to MACI that needs the synergy of invasive surgery and is limited by donor sites, stem cells show greater potential to treat various cartilage diseases in a minimal-invasive manner due to their available clinical sources and multiple compelling functions. However, the low targeting efficiency, impaired cell viability and function, and unutilized cell differentiation into cartilage lineages after in vivo transplantation resulted in the use of stem cells in high dose but the relatively poor therapeutic effects, which substantially limit the application of stem cell therapy [21–23]. Therefore, the target delivery and precise regulation of stem cells and/or other bioactive substances is promising to improve the outcomes of various MSDs.

Microrobots, the micromachines in sizes of nanometers to submillimeters, have been developed for the minimal-invasive, targeted, intelligent, and adaptable delivery of stem cells and other drugs, which facilitates to improve their therapeutic efficiency and avoid adverse side reactions [24]. Initially inspired by motile microbes, the intention of microrobots was to move to the root site of a disease to deliver the appropriate drugs/cells [24]. The actuation of microrobots is generally achieved by converting different forms of energy into mechanical energy. These include chemical actuation (hydrogen peroxide (H_2_O_2_)-based and enzyme-based reactions) [25–28], physical actuation (magnetic, optical, electrical, and acoustic actuation) [29–34], and biological actuation (bacterial/eukaryotic cell-based actuation) [35–38]. Active motion allows microrobots to reach the target position efficiently, which endows them the potential to revolutionize minimally invasive medicine and targeted therapies. With the continuous advancement of nanotechnology, materials science, and control engineering, as well as the trend of miniaturization, precision, and intelligence in medical technology, microrobots show great potential for precise drug delivery in treating various diseases [39]. The advantages of microrobots in the musculoskeletal system mainly involve minimal-invasive intervention, precise delivery, real-time monitoring, and remote regulation.

**Fig. 1:**
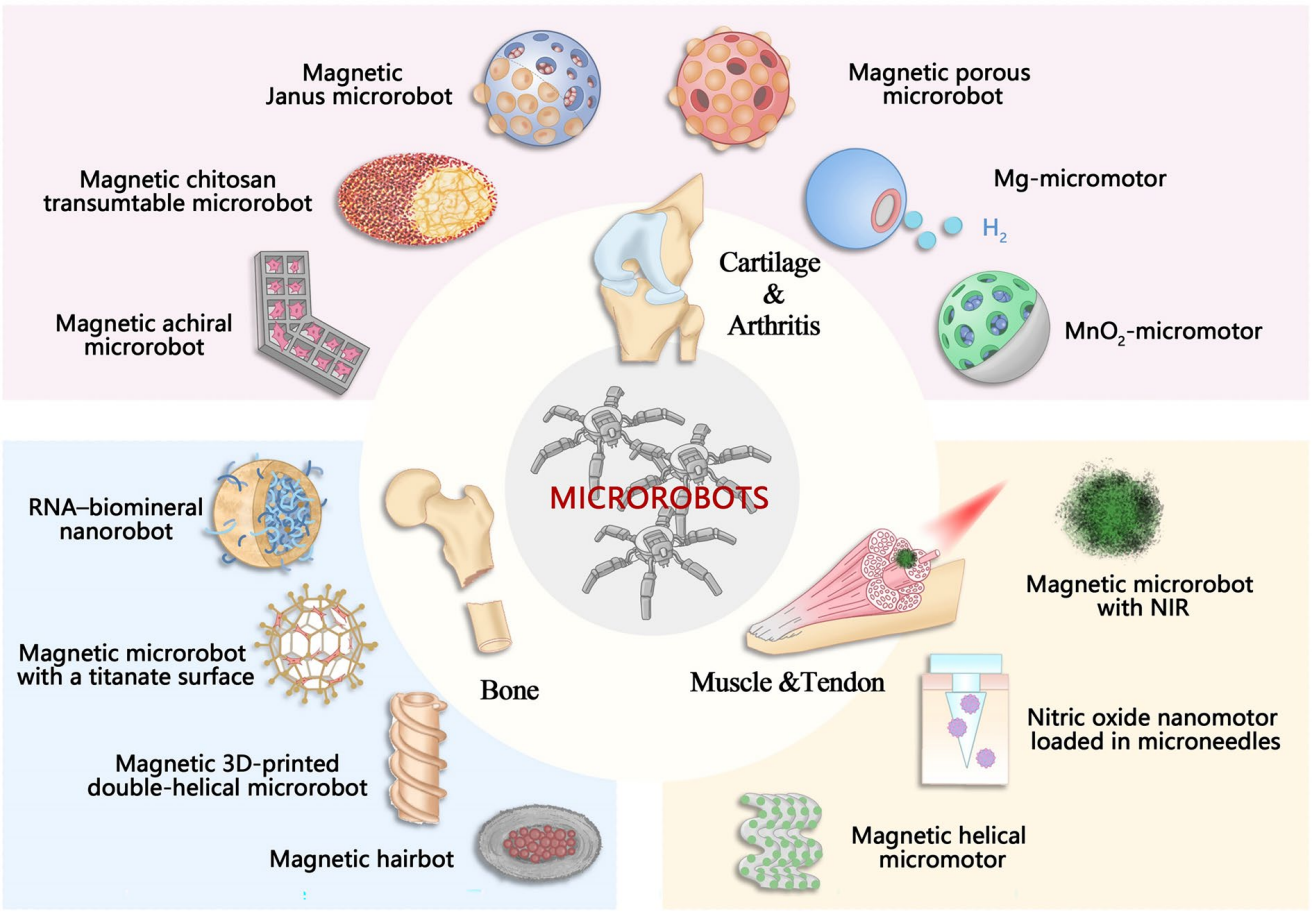
The characteristics of microrobots applied in the musculoskeletal system. The microrobots used to treat bone, cartilage, muscle, and tendon diseases are illustrated

Currently, the main functions of microrobots in the musculoskeletal system include the following two aspects: (1) as a stem cell/drug delivery system, delivering exogenous cells or regulating endogenous cells to exert regenerative functions through precise targeting [40]; (2) as a "scavenger" of tissue damages, improving the pathological microenvironment of injured tissues by converting harmful substances into beneficial ones [41]. Figure 1 displays the characteristics of microrobots applied in the musculoskeletal system.

Nevertheless, there are still many challenges in the application of microrobots in vivo. The first and foremost requirement is to apply biosafe materials to fabricate microrobots [42, 43]. In addition, microrobots tend to motor in swarms, and how to drive a swarm of microrobots through a precise control system is also an urgent challenge to be solved [44]. Finally, given that most current microrobot research is conducted in vitro environments and under optical microscopes, which are quite different from clinical reality. Consequently, it is necessary to develop appropriate, clear, real-time medical imaging techniques to localize and track microrobots [45]. Besides, it is the small size of microrobots that makes their fabrication, control, and imaging quite difficult [46]. In the future, the treatment of MSDs will gradually move toward minimally invasive, prevention-oriented, fine-tuned, and targeted therapies. These directions can be quite compatible with the advantages of microrobots, which probably solves the current MSD therapeutic dilemma.

In this review, we will first introduce the characteristics of musculoskeletal system and regenerative medicine. This will be followed by a description of common musculoskeletal diseases, treatment strategies, and current challenges. In addition, recent advances in microrobots applied in the musculoskeletal system are comprehensively discussed. In the following section, we summarized the development of actuation and imaging systems that are integrated with microrobots for precise control, real-time monitoring, and postoperative tracking. Finally, the limitations and challenges of current microrobots used in the musculoskeletal system and future development of microrobotic systems are concluded.

## Musculoskeletal System and Regenerative Medicine

The musculoskeletal system consists of bones, muscles, cartilages (articular cartilage, intervertebral disks, and meniscus), tendons, and other connective tissues that provide stability to joints [47]. These tissues form a complex structure that not only supports body weight and protects internal organs, but also precisely controls and maintains the functions of body movements at the macroscopic level [48]. At the microscopic level, these tissues also have endocrine functions which are mainly reflected in their roles in humoral signaling and energy supply, which together affect the homeostasis of organism [49–52]. Worldwide, MSDs have become the second most crucial factor leading to disability and have increased by nearly 20% over the past decade, which primarily involves the injuries of cartilage, tendon/ligament, bone, and skeletal muscle [53].

### Common Diseases and Treatment Strategies in the Musculoskeletal System

Articular cartilage is a highly connective tissue that functions between bones to provide lubrication, reduce friction, and decompose forces with the goal of preventing intra-articular abrasion and facilitating motion. Articular cartilage injury and OA are common clinical orthopedic diseases, which are also the primary causes of chronic disability in middle-aged and elderly people [54]. According to the World Health Organization, about 9.6% of men and 18% of women over 60 years of age around the world suffer from cartilage lesions, leading to enormous medical costs and heavy social burdens. Due to the lack of vessels and nerves, articular cartilage shows an extremely low regenerative capacity, and there are no available drugs or treatments to effectively delay or reverse the progress of cartilage injuries and OA. Generally, in the middle and late stages of OA, the efficacy of conservative treatment like NSAID and intra-articular hyaluronic acid (HA) injection is very limited, and patients with severe symptoms tend to require surgery treatment such as joint replacement [55, 56].

Tendons and ligaments are dense connective tissues that connect bone to muscle or the other bone for force transmission. Tendons and ligaments show a high similarity in component, structure, and function, and thus both of them are referred as “tendon” to discuss in the following sections. Similar to cartilage, tendons lack blood vessels or nerves, thereby presenting a poor capacity of self-healing [57, 58]. Although tendons are one of the strongest and most fatigue-resistant tissues in human body [59], overuse, aging, metabolic diseases, and other risk factors disturb tendon homeostasis and result in the development and progress of tendinopathies, manifested as persistent pain and impaired joint function [60–62]. Tendon disorders account for 30%–50% of MSDs, resulting in a decreased life quality of individuals and a huge social burden [53, 63]. Conservative treatments like NSAIDs and physiotherapy are widely used for pain relief and inflammation alleviation for acute and chronic tendon disorder and surgical treatment including suturing and autograft/allograft/artificial graft implantation is needed for mass or complete tendon ruptures [57, 64]. However, current treatments for tendon disorders are associated with poor outcomes and various complications, including tendon adhesion, scar formation, re-rupture, and muscle atrophy [65–67].

Bone is a stiff and complex tissue that constitutes skeletal system to provide structural support, posture maintenance, protection of internal organs, and storage of minerals. Fracture represents a significant category of bone injuries, characterized by the disruption of bone continuity when bone is exposed to mechanical forces surpassing its strength. Most of fracture could be perfectly repaired after being appropriately fixed due to the strong reparative capacity of bone tissues. However, the homeostasis and self-healing capacity of bones is substantially impaired in various pathological conditions, such as osteoporosis and Paget's disease [68]. In trauma orthopedic wards, nonunion or delayed healing occurs in 5%–10% of fractures due to impaired fracture healing and bone defects [69]. Autografts, allografts, xenografts, and biomaterial fillers are effective to treat nonhealing and delayed healing fractures, while the limited donor tissues, potential immune injection, and possible infection risk hinder their widespread applications [70, 71].

**Fig. 2:**
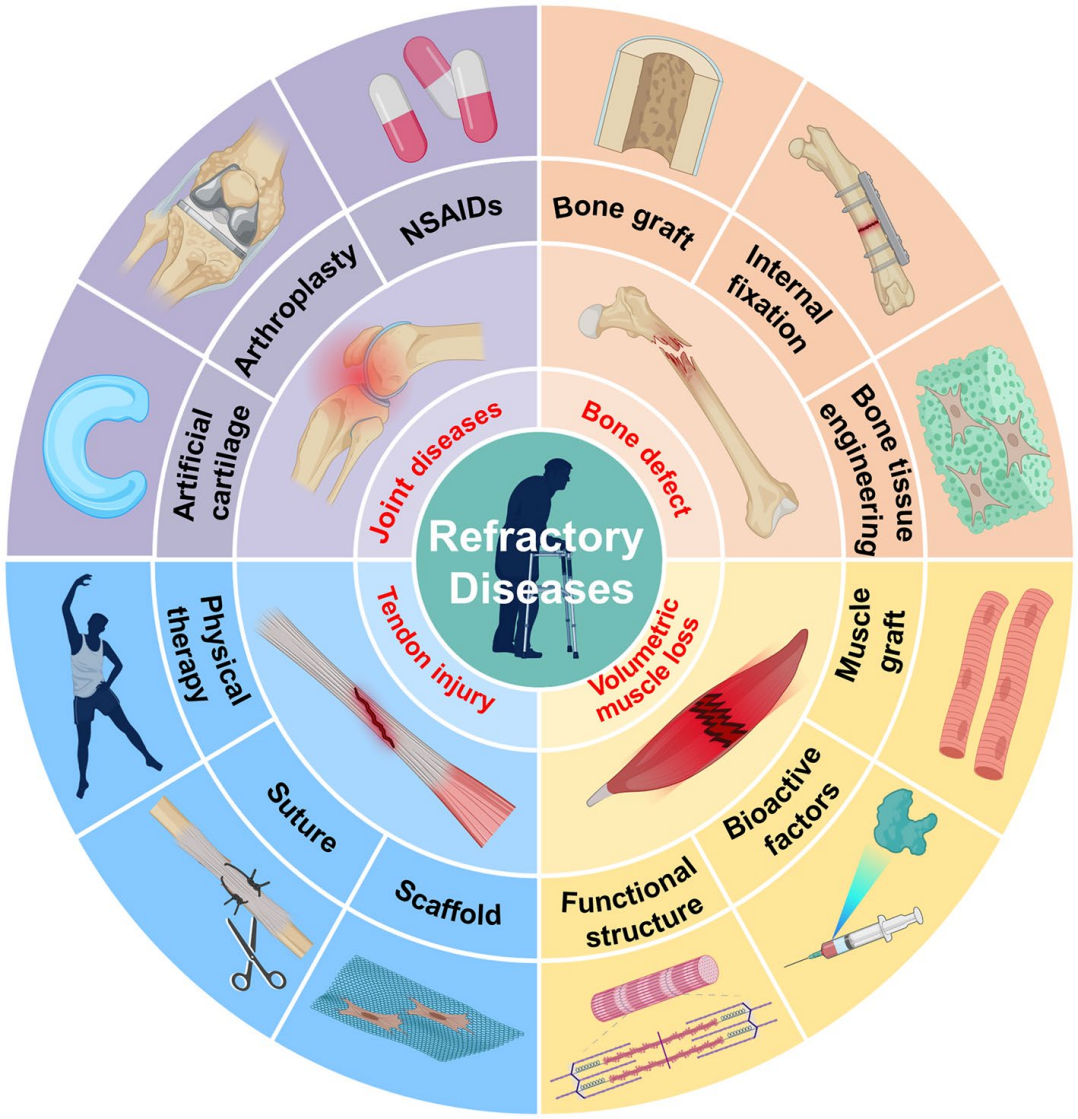
Outline of the four refractory diseases of the musculoskeletal system and their current treatments. Notes: NSAIDs, nonsteroidal anti-inflammatory drugs

Skeletal muscle is the most abundant soft tissue that produces contractile forces to drive body movement [72]. Skeletal muscle is located superficially in the body and usually bears the brunt of injury when trauma strikes [73]. Owing to the strong regenerative capacity of muscle stem cells, often referred to as "satellite cells," minor muscle injuries are generally self-healing [74]. However, severe trauma or surgery-induced muscle loss exceeds the endogenous self-repair capacity of muscle and is referred as volumetric muscle loss (VML) [75, 76]. VML induced chronic inflammation, massive loss of satellite cells, and extensive fibrosis, resulting in long-term dysfunction and permanent disability [73, 77]. The current gold standard for the treatment of VML is functional muscle tissue transplantation [78]. However, muscle transplantation is also restricted by the limited structural and functional recovery, low tissue availability and high donor site morbidity [79, 80]. Figure 2 shows the four refractory diseases of the musculoskeletal system and their current treatments.

### Challenges and Potential Roles of Microrobots in Treating MSDs

Due to the distinct tissue properties in the musculoskeletal system, the strategies to treat MSDs are quite different, with the self-healing ability of specific tissue considered [12, 81, 82]. For skeletal muscles and bones with the strong reparative capacities, minor injuries are usually left to repair with conservative treatments like rest, physiotherapy, and NSAIDs, and the larger injuries that do not far exceed their self-regenerative capacity can be well treated with the synergy of sample surgical operations, such as suturing and internal fixation [83–85]. In contrast, for cartilages and tendons that lack blood vessels and nerves and are less capable of self-repair, even small injuries are possible to cause severe outcomes if proper interventions are not carried out at an early stage. Most of current conservative and surgical treatments for these tissues also exhibits poor effects on relieving symptoms, recovering structure and functions, and rescuing disease progression, with the high risk of dysfunctions and complications. Ultimately, end-stage surgery like autograft/allograft transplantation and arthroplasty is inevitably required to restore and reconstruct the impaired cartilages and tendons [56, 57]. In addition, end-stage surgery is also used to treat severe or pathological injures of skeletal muscles and bones that far exceed their self-regenerative capacity. With the emergence of early intervention concepts and regenerative medicine, scientists and clinicians are committed to precisely recognize and minimal-invasively treat refractory MSDs in the early stage to improve therapeutic outcomes and avoid terminal surgery.

To achieve these goals, the precise and controlled delivery of various “drugs” like stem cells, anti-inflammatory agents, growth factors, biomaterials, and genes to improve the pathological environment or enhance the regenerative ability of injured tissues is critical. Characterized by the remarkable capacity of self-renewal, multilineage differentiation, immune modulation, stem cells have emerged as the multifunctional “drug” that could intelligently respond to the pathological microenvironment and initiate different regenerative processes in the musculoskeletal and the other systems [22]. Of all stem cell populations, the most widely used and promising are mesenchymal stem cells (MSCs), which can be derived from various tissues, such as bone marrow [86, 87], adipose tissue [88], umbilical cord [89–91], peripheral blood [92], teeth [93], and urine [94]. Some of these stem cells sources like umbilical cord and the surgically extracted adipose tissues or wisdom teeth were often trashed as medical waste, and thus could be easily obtained. Clinical trials have demonstrated the safety and efficiency of stem cell injections with or without biomaterials on treating various refractory MSDs, including OA [95–100], rotator cuff injury [101], bone nonunions [102], and Achilles tendinopathy [99, 103]. Although current stem cell therapy displayed a positive role in pain relief and functional recovery, its therapeutic effects are far away from the expected ones, which could be attributed to the low targeting efficiency, decreased cell viability, unutilized differentiation ability after in vivo transplantation [21–23]. These limitations cause the phenomenon that a high dose and repeated injection of stem cells is required to ensure the therapeutic efficacy, hindering the widespread use of stem cell therapy [98]. Therefore, the targeted delivery and precise regulation of stem cells or other bioactive substances in minimal-invasive manner is critical to effectively treat refractory MSDs in the early stage.

Recent advances in microrobots-based delivery system are promising to overcome the current limitations of drug/cell delivery in treating MSDs. The microrobots could be fabricated by biocompatible and biodegradable materials like chitosan, collagen, and alginate that were widely demonstrated to provide 3D microenvironment to support various cell behaviors and functions, thus effectively maintaining a high cell viability and stemness after in vivo transplantation of stem cells [104, 105]. More critically, microrobots in the synergy of actuation and imaging system could precisely deliver and real-timely monitor the transplanted stem cells and drugs to target the lesions or diseased sites of musculoskeletal system [106, 107]. Various noninvasive manipulations based on magnetic field, light, and ultrasounds and multifunctional microrobot designs endow microrobots active motion ability and make it possible to remotely control the function and lineage specification of stem cells, as well as the interactions of microrobots and cells with the local pathological microenvironment [106–108], which is critical for the endogenous or exogenous cells to initiate reparative processes. In addition, the microscale or nanoscale of microrobots also allows for the minimal-invasive delivery of stem cells and other drugs, showing a great potential in treating refractory MSDs.

## Microrobots Applied in the Musculoskeletal System

### Design of Microrobots in the Musculoskeletal System

To realize the minimal-invasive, targeted, intelligent, and adaptable delivery of stem cells and other drugs for precise MSD treatment, several basic components of microrobots should be considered, including core materials, actuation/navigation system, and imaging/tracking system [24]. The core materials refer to the biomaterials to fabricate or form microrobots, i.e., the skeleton of microrobots. First, the core materials not only must be biosafe for in vivo application but also be responsive to stimulations that endow microrobots with the ability of active motions to target lesions [24]. Specifically, biocompatible and biodegradable materials like chitosan and alginate should be used to prepare microcarriers in micro- or nanoscale that support the loading and release of drugs, and could degrade harmlessly in the body [105]. For the cell-delivered microrobots, a proper 3D microenvironment also should be constructed for the adhesion, viability, proliferation, and functional maintenance of stem cells [105]. In addition, the incorporation of biomaterials that respond to external stimulation (e.g., magnetic field, light, and ultrasound) and internal environments (e.g., ROS and inflammation) is essential for the active, intelligent, and adaptive delivery with or without the guidance of actuation systems [106, 107]. Second, microrobots alone are difficult to autonomously and precisely deliver drugs to the targeted area, and thus usually are integrated with an actuation system [106, 109]. For instance, electromagnetic actuation systems (EMA) can generate rapidly varying and complex fields like gradient, oscillating or rotating fields to drive the diverse locomotion of microrobots [110]. Last, to improve the accuracy and efficiency of drug delivery using microrobots, an imaging system, such as Doppler ultrasound, is recommended to be introduced for the real-time monitoring of microrobots after in vivo injection, especially for the swarm manipulation of microrobots in a 3D complex environment [111, 112]. The tracking of microrobots after targeted delivery is also important, which could confirm the retention or fixation of microrobots into the defects to repair tissues. The magnetic or fluorescent microrobots could be tracked by current medical imaging techniques, including X-ray computed tomography (CT), magnetic resonance imaging (MRI), and fluorescence imaging. In addition to these basic components of microrobots, more attentions should be attached to the tissue properties, pathological environment, and application scenarios to improve the therapeutic effects of microrobot-based drug delivery and biological regulations.

### Application of Microrobots in the Musculoskeletal System

**Fig. 3:**
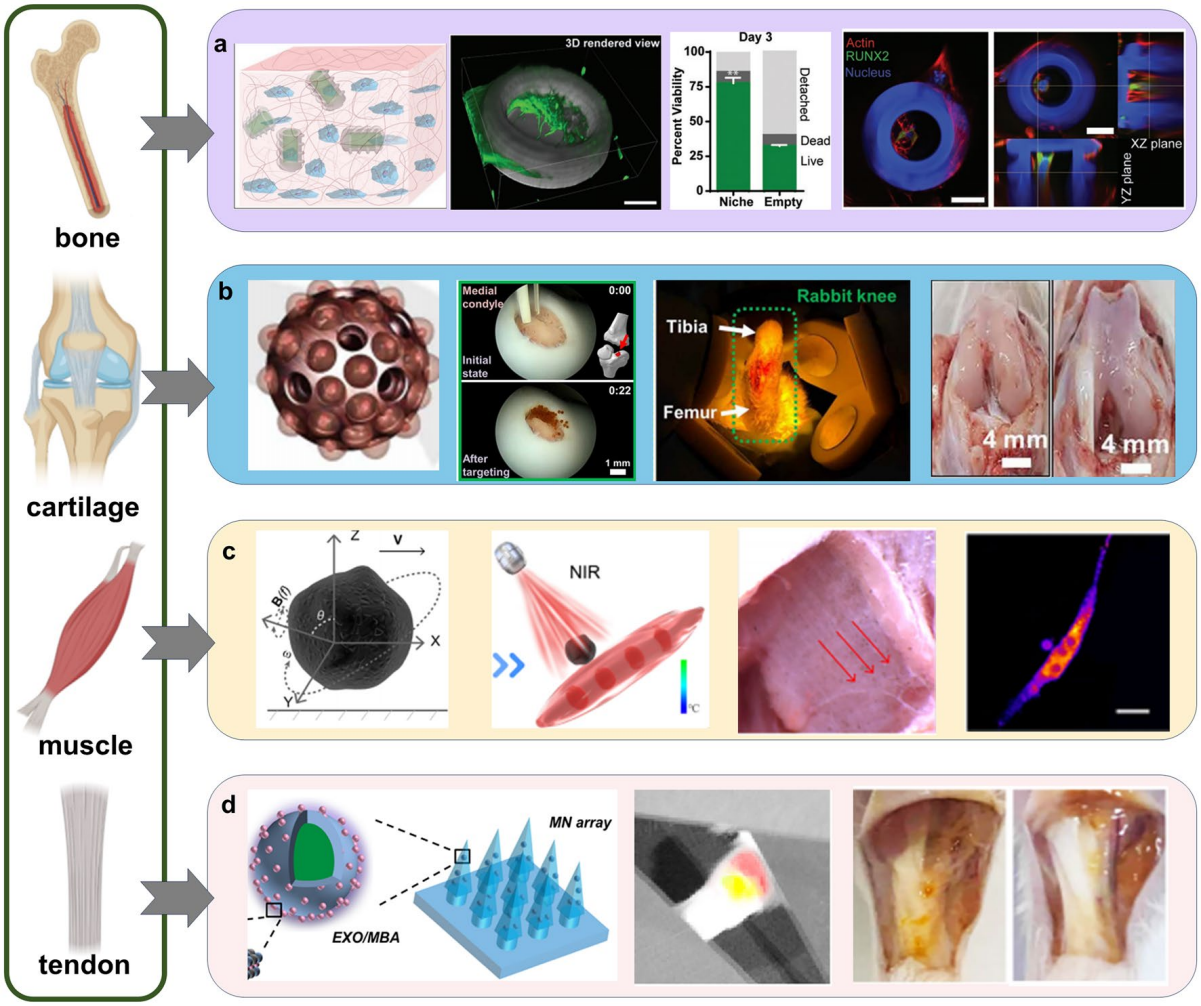
Typical applications of microrobots in the musculoskeletal system. **a** Spiral MCT improved the viability and osteogenic differentiation of stem cells [161]. Copyright (2019) John Wiley and Sons. **b** Targeted delivery of porous spherical microrobots to repair cartilage defects in vivo under arthroscopy [106]. Copyright (2020) American Association for the Advancement of Science. **c** Microswimmers for precise muscle stimulation in the presence of magnetic fields and NIR [181]. Copyright (2022) American Chemical Society. **d** Nanomotors loaded in microneedles improved the therapeutic efficiency of EXOs in Achilles tendinopathy [108]. Copyright (2021) American Chemical Society

The tissues of musculoskeletal system work together to support the body and maintain motor function, and they are both interconnected and independent of each other. Different tissues have distinct repair mechanisms and self-repair capabilities, and thus the focus of microrobots in each tissue differs and needs to be discussed separately. Figure 3 illustrates typical applications of microrobots in the musculoskeletal system.

#### Microrobots for Cartilage Repair

**Fig. 4:**
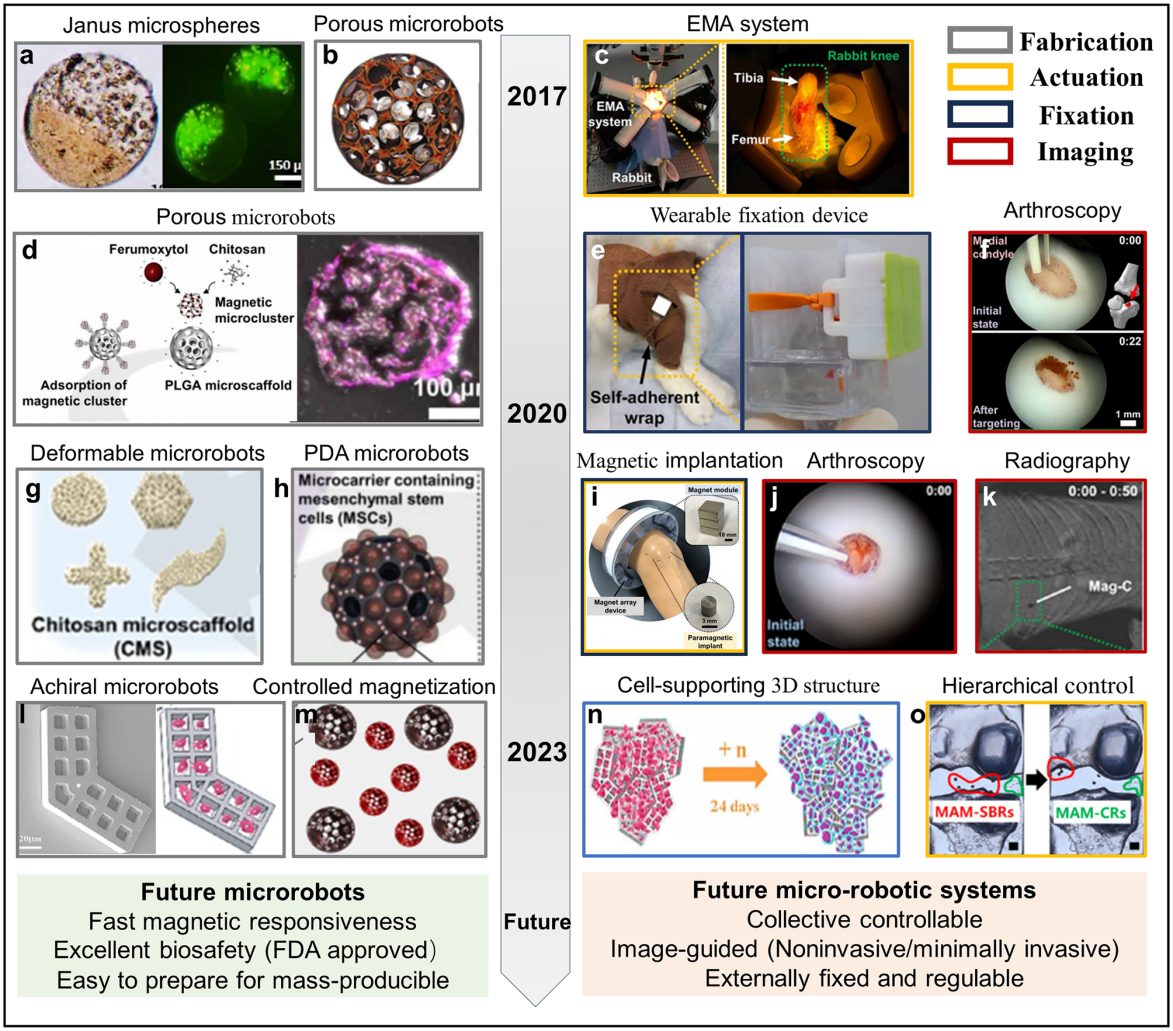
The development and future prospects of microrobot systems applied in cartilage repair. **a** Janus microspheres encapsulated half with stem cells and half with magnetic particles [104]. Copyright (2018) Elsevier. **b** Magnetic PLGA microrobots [109]. Copyright (2017) John Wiley and Sons. **c** EMA system applied to cartilage targeting in rabbits [106]. Copyright (2020) American Association for the Advancement of Science. **d** Schematic and confocal images of a porous microrobot with stem cells [106]. Copyright (2020) American Association for the Advancement of Science. **e** Wearable magnetic device fixed on the rabbit's knee and a phantom [106, 120]. Copyright (2020) American Association for the Advancement of Science, MDPI. **f** Targeted delivery of microrobots to cartilage defects under arthroscopic guidance [106]. Copyright (2020) American Association for the Advancement of Science. **g** Microrobots with programmable morphology for different application scenarios [105]. Copyright (2020) American Chemical Society. **h** PDA-coated microcarriers [122]. Copyright (2021) John Wiley and Sons. **i** The wearable magnet array device consisting of magnet modules [122]. Copyright (2021) John Wiley and Sons. **j** Arthroscopic-guided microrobot delivery [105]. Copyright (2020) American Chemical Society. **k** X-ray-guided microrobot delivery [105]. Copyright (2020) American Chemical Society. **l** 2D nonchiral waffle-shaped microswimmers [134]. Copyright (2023) American Chemical Society. **m** Microrobots with different sizes and magnetizations for cartilage and subchondral bone repair [125]. Copyright (2023) MDPI. **n** Microswimmers assemble to form cell-supported 3D structures [134]. Copyright (2023) American Chemical Society. **o** Microrobots moved sequentially to the subchondral bone and cartilage defects [125]. Copyright (2023) MDPI

With the emergence of tissue engineering and regenerative medicine, researchers have attempted to deliver drugs, bioactive factors, or stem cells with or without biomaterials to improve the pathological microenvironment of endogenous cells and enhance the regenerative ability of injured articular cartilages, thereby promoting cartilage repair in the early stages and avoiding terminal surgery. For example, the articular injection and biomaterial-based delivery of MSCs show the potential roles in relieving pain, promoting cartilage repair, and improving joint function in patients with OA or cartilage defects [21, 113]. However, current MSC therapies presented very limited effects on promoting cartilage repair, which could be attributed to the low and short-lasting cellular viability, unutilized differentiation ability, and low targeting efficiency of MSCs in joints [21]. How to minimal-invasively, precisely, and intelligently deliver stem cells or other bioactive substances to effectively improve cartilage repair remain great challenges. In recent years, various microrobots with the features of minimal invasion, active motion, multifunctionality, high safety, and adaptivity have been designed as cargo-carrying micromachines to move to the site of lesion or disease [24], which sheds light on the treatment of cartilage diseases, which is shown in Fig. 4.

The team of Eunpyo Choi and Jong-Oh Park performed a series of studies to develop magnetic microrobots, EMA system, and wearable or implantable medical devices to deliver stem cells for chondral and osteochondral repair and achieved many promising results. In 2017, this team first designed a magnetically actuated microrobots, a spherical and porous microscaffold consisting of poly (lactic-co-glycolic acid) (PLGA) and polyethyleneimine (PEI)-coated magnetic nanoparticles (MNPs), for targeted delivery of MSCs (Fig. 4b) [109]. In vitro experiments revealed that the MSCs loaded into microrobots showed a high viability and normal proliferation, and could differentiate into cartilage lineage after chondrogenic induction. In addition, the microrobots had a saturation magnetization of 8.105 emu g^−1^ and coercivity of near-zero, with a similar superparamagnetic behavior to PEI-coated MNPs. Thus, these magnetically responsive microrobots could be precisely manipulated in 3D space, and displayed the increased moving velocities from ~ 100 to ~ 200 μm s^−1^ in a simulated synovial fluid under the gradient magnetic fields of 0.9–1.8 T m^−1^ (interval 0.3 T m^−1^) produced by an EMA system. In targeting tests, the delivery efficiencies of microrobots were 93.3% in the 2D chamber and 100% in the 3D knee joint model, respectively (Fig. 4c). Besides, MSCs could not be lost from the microrobots during the magnetic guidance. These findings confirmed that the developed microrobots in combination of EMA system were promising to perform targeting stem cell delivery for articular cartilage repair. In general, this study developed a preliminary microrobotic system including microrobots, actuation system, and imaging system, which offered a proof of concept for precisely delivering stem cells by microrobots to target articular cartilage lesions. However, how the targeted microrobots remain in lesions to initiate regenerative processes; whether the delivered MSCs can effectively repair the injured cartilages, and if the microrobot delivery system could approach clinical translation are not shown in this study. Subsequently, another study by this team answered the above questions (Fig. 4d) [106]. For the sake of approaching clinical applications, this work optimized the microrobot system with more consideration of microrobot material safety, magnetic fixation, and clinical safety and efficiency [114]. First, the authors chose clinically safe and biodegradable ferumoxytol, an FDA-approved superparamagnetic iron oxide nanoparticle used as a magnetic resonance imaging agent [115]. Since the strong negative ionic charge and limited surface area of the porous PLGA microspheres inhibited the absorption of ferumoxytol, chitosan, an FDA-approved cationic polysaccharide with excellent biocompatibility and degradability [116], was introduced to fabricate microrobots [117]. The developed microrobots exhibited an average velocity of 2.88 mm s^−1^ under a constant magnetic field of 30 mT and gradient of 0.60 T m^−1^. Second, the magnetic fixation device, a single magnet, was added to immobilize microrobots in the lesions after the targeted delivery (Fig. 4e). The feasibility and efficiency of microrobot fixation were evaluated on porcine cartilage samples in vitro. The results revealed that microrobots were efficiently fixed inside the cartilage defects at a physical shaking of 50 rpm, with a substantially increased fixation efficiency of 97% compared with no magnet one (68%), which was further validated in a rabbit cartilage defect model (Fig. 4f). Critically, the authors first evaluated the effects of targeted stem cell delivery by using this microrobot system on cartilage repair in vivo. The MSCs delivered by microrobots could survive at least three weeks after in vivo transplantation and promote hyaline cartilage formation. The results also revealed that the MSCs-loaded microrobots did not significantly activate host immune responses after articular injection, presenting a high biocompatibility and biosafety for clinical application. Generally, this study successfully constructed a complete microrobot system that consists of magnetic microrobots, EMA system, fixation devices, and imaging system, and verified the feasibility of targeted MSCs delivery by using this microrobot system to initiate cartilage repair. It is the first study to deliver stem cells by using a microrobot system to simulate cartilage repair in vitro and in vivo, so it had important implications. However, it was worth noting that PLGA microscaffolds could degrade into lactic and glycolic acids, which were reported to cause cartilage damage [118]. In animal experiments, 100 microrobots could only deliver about 8 × 10^4^ MSCs in 4 h, which was much less than the used cell amounts (8 × 10^5^ to 8 × 10^7^ cm^−2^) in previous clinical trials [119]. Therefore, to confirm the superiority of the microrobotic delivery system, it would be more convincing to perform comparative experiments with the injection of MSCs or MSCs-loaded hydrogels in future clinical trials.

In recent years, the team of Eunpyo Choi and Jong-Oh Park continued to perform studies to improve this microrobot system and explore its potential applications. One of these works improved the fixation strategy of microrobots by designing a wearable magnetic device whose core is an optimized array of permanent magnets based on the Halbach magnet principle [120]. This device could provide a strong and concentrated magnetic field for microrobot fixation at the desired locations and showed higher potential for clinical application compared to a single magnet [121]. To overcome the limitations of the EMA system like large installation and workspace, high energy consumption, low accessibility of medical staff, and improve the chondrogenic differentiation of stem cells for cartilage repair, the study of Gwangjun Go developed a multifunctional magnetic implant system consisting of a biomaterial-based microrobot containing TGF-β, a biocompatible paramagnetic implant, and a portable and wearable magnetic array device (Fig. 4h, i) [122]. In this study, a single microrobot could load ≈1.52 ng TGF-*β* and 3.3 × 10^3^ of stem cells, and the sustained release of TGF-*β* simulated the chondrogenic differentiation of stem cells. The biocompatible paramagnetic implant was a parylene-coated cylindrical pure iron, which could be magnetized by an external magnetic field provided by a magnet array device. Interestingly, multiple paramagnetic implants could be transplanted into a large area of the femur simultaneously, and the targeting and fixation of microrobots could be finished by the proposed magnetic carrier system in coordination with the wearable magnet device, presenting a high targeting efficiency of over 90% in a phantom model mimicking femoral condyle defect. Despite these advantages, this system may not be clinically feasible because it does not fit the concept of minimal invasion, and the transplantation of paramagnetic implants probably caused subchondral bone damage [123]. In clinical, the defects of articular cartilage often extend to subchondral bone with the progress of OA and trauma-related cartilage injuries and thereby form osteochondral defects, the most severe type of cartilage injuries [124]. Ingeniously, this team achieved a one-step targeted delivery of different stem cell-loaded microrobots for osteochondral repair by merely controlling the size of magnetic microrobots [125]. Since the pore size of scaffolds that facilitated for osteogenesis was higher than that for chondrogenesis [126], the authors fabricated magnetic microrobots with a size of 285.09 ± 17.38 µm and pore size of 63.82 ± 9.91 µm for cartilage regeneration (MAM-CR) and those with a size of 771.09 ± 46.4 µm and pore size of 79.16 ± 11.00 µm) for bone regeneration by changing the concentrations of PLGA and gelatin (Fig. 4m). Due to the different sizes of microrobots, the absorbed MNPs to the surface of porous PLGA microspheres were different, which resulted in that the moving speed of MAM-SBR was three times higher than those of MAM-CR under the same magnetic field [127]. Therefore, MAM-SBR and MAM-CR could be successively actuated to the subchondral and chondral layers of osteochondral defects under the guidance of the same EMA system, showing an attractive application of microrobot-based stem cell delivery in treating osteochondral injuries (Fig. 4o).

As mentioned above, the degradation products of PLGA scaffolds, like lactic and glycolic acids, may induce inflammatory responses and cause cartilage damage [128]. Therefore, to avoid these side reactions and improve clinical safety, the FDA-approved and biodegradable chitosan was used to fabricate magnetic microrobots (Fig. 4g) [129]. In the microrobot preparation, porous chitosan sheets were firstly prepared via freeze-drying, and then were quickly and precisely manufactured into the microscaffolds with desired shapes and motions by using a femtosecond-pulsed UV laser cutting machine [105]. In vitro experiments demonstrated that the hADSCs loaded in microrobots showed a high cell adhesion and viability, and were able to differentiate into chondrocytes, confirming the compelling compatibility of chitosan microrobots [130]. Overall, this study presents a magnetic chitosan microrobot with high biosafety, which is more available for clinical applications than PLGA microrobots (Fig. 4j, k). Similarly, Ma et al. prepared a magnetic chitosan microrobot through anti-phase suspension and phase separation of chitosan and in situ polymerization of Fe_3_O_4_ with dopamine [131]. The magnetic microrobots exhibited a paramagnetic property for magnetic responsiveness, suitable porosity, and pore size for the growth of bone marrow mesenchymal stem cells (BMSCs) and good biocompatibility. When implanting the BMSCs-loaded microrobots into the cartilage defects of rats, the microrobots could be fixed into cartilage defects by using magnet fixation device, and finally promoted cartilage repair. Gait analysis further confirmed that the application of this microrobot system effectively reduced postoperative pain and promoted functional recovery in rats. The study of Thomas et al. developed magnetic Janus alginate microspheres to serve as microrobots (Fig. 4a) [104]. Alginate is a natural polymer derived from seaweed that protects cells from host immune responses [132]. Notably, Janus microspheres minimize the toxicity of iron oxide nanoparticles (IONs) to cells by encapsulating IONs and MSCs in separate compartments, as indicated by the high cell viability of MSCs on microspheres. This design was also used to load nanodrugs to exert cell-drug synergy [133]. However, this study only evaluated the biocompatibility and magnetic responses of microspheres without in vivo tests, so there is no way to evaluate their ability to repair cartilage.

Recently, a study by Chen et al. proposed a waffle-shaped, Ni and Ti-coated, and 2D porous silicon microswimmer that could swim in liquids and roll on surfaces in a 3D environment when controlled by a rotating magnetic field (Fig. 4l) [134]. The waffle shape was used because a top angle close to 120 degrees facilitated the best swimming speed [135]. Similar to previous porous microrobots, the microswimmer had multiple square microwells to load cells [136], and the depth of each well was designed to be 25 μm to ensure stem cell adhesion and proliferation and be a nonchiral planar shape to allow for cell retention [137]. The research team explored the movement patterns of microswimmers in different fluids, and found that their rolling speed was always greater than their swimming speed at the same frequency, which indicated that rolling motion was suitable for the long-distance travel while swimming motion could be adopted to overcome complex terrains. They further verified the ability of microswimmers to overcome complex terrains via switching the motion modes. The results revealed that microswimmers in artificial cerebrospinal fluid could get close to the target at high rolling speed (~ 304 μm s^−1^ at 5 Hz) on a flat surface and subsequently switch to swimming with a maximum velocity of ~ 72 μm s^−1^ at 7.5 Hz to overcome obstacles or inclined pathways to reach the target, which was further validated in a 3D knee model. Finally, the microswimmer could also be stacked like building blocks and in situ assembled into 3D structures for cell culture at the site of cartilage defects (Fig. 4n). Despite the use of cytotoxic nickel/titanium coatings rather than IONs, it did not affect the proliferation and chondrogenic differentiation of BMSCs [138]. In general, this study developed a novel 2D microrobots with rolling and swimming motions for targeted cell delivery and 3D cell culture in the lesion sites, which made the application of microrobots in the knee joint more feasible.

In addition to cartilage damage, rheumatoid arthritis (RA) is also a refractory disease around the knee joint [139, 140]. Excessive ROS increase is closely related to RA development [141, 142], and hydrogen not only scavenges oxygen radicals but also shows significant anti-inflammatory effects [143, 144]. Therefore, Xu et al. developed a self-propelled magnesium-HA nanomotor, which could continuously supply active hydrogen to attenuate localized oxidative stress and accurately self-propel to the inflammatory region under the guidance of ultrasound [107]. The source of power for the self-driven nanomotor was hydrogen generated by redox reactions between magnesium and body fluids, and the nanomotor could move in linear or helical motion in body fluids [145]. Owing to the HA hydrogel-PLGA coating, the nanomotor could stay in the body for about 5 min, which greatly increased hydrogen production. In addition, the generated bubbles also facilitated the real-time monitoring of the nanomotor by ultrasound. The nanomotors showed a more effective role in scavenging ROS and inhibiting inflammatory responses compared to conventional drug therapies [146]. From the disease mechanism of RA, this study designed a self-actuated nanorobot to improve the pathological environment by removing excessive ROS and inhibiting pro-inflammatory responses, thus inhibiting the progress of RA [147]. Besides, a complete, closed-loop, and effective microrobot system was constructed with the integration of actuation, therapy, and imaging for future clinical application. Recently, another study by Xu et al. continued to work on the mechanism, and it turned its attention to H_2_O_2_, a kind of ROS that caused tissue damage and chronic inflammation [41, 148, 149]. In this study, a MnO_2_-motor, consisting of ceria nanoparticles and a MnO_2_ shell, was developed to scavenge H_2_O_2_ and generate oxygen which ultimately inhibited the pro-inflammatory phenotype of macrophages [41]. In addition, fueled by H_2_O_2_ at 10 mM in PBS and 1 mM in simulated synovial fluid, the MnO_2_-motor showed an average fusion speed of 4.3 and 3.4 μm s^−1^, which supported the rapid diffusion of the MnO_2_-motors in H_2_O_2_-abundant microenvironment for ROS scavenging and inflammation attenuation. Ultimately, the intra-articular injection of MnO_2_-motors was demonstrated to substantially attenuate hypoxia, synovial inflammation, cartilage degradation, and bone erosion, and thereby successfully alleviated the progression of RA [150]. It was believed that these microrobots would be more promising in RA treatment with further improvements in material, actuation, and functional designs.

In summary, various microrobots, like magnetic spherical/Janus microspheres, magnetic microswimmer, and Mg/MnO_2_-micromotor, have been developed to precisely deliver stem cells to the targeted cartilage areas or efficiently improve the pathological environment of injured cartilages for promoting the repair of articular cartilage and osteochondral defects (Table 1). As for the microrobots with passive motion, several actuation and fixation systems of microrobots were also designed to increase the efficiency of targeted delivery and cartilage repair, including EMA system, magnetization implant system, and wearable magnetic device. Among these systems, the EMA system equipped with an operating microscope, fixation device, and imaging instrument shows the greatest potential for future clinical applications. Importantly, the application of microrobots and microrobotic systems has shown compelling results in increasing the targeting efficiency of stem cell delivery, improving the pathological environment of injured cartilages, and promoting the structural and functional regeneration of cartilage defects in small animals like rats and rabbits. However, further studies based on large animal and clinical trials are urgently needed to approach the clinical translation of the microrobot system.

**Table 1:** Summary and comparison of the characteristics of microrobots in the musculoskeletal system

Objectives	Type of microrobots	Actuation	Advantages	Disadvantages	References
Bone regeneration	Variable-stiffness actuators	MF	Variable stiffness for bone development Bioinduced programmable mineralization	Unknown biocompatibility	[154]
	RNA nanomachines	CR	Simulating bone healing on the nanoscale in vitro Rapid and controlled collagen mineralization	Dependent on collagen scaffolds for delivery	[167]
	Hairbots	MF	Simple preparation, low cost, and rapid mass production Medullary cavity for drug loading and ultrasound imaging	Unclear degradation Low efficiency of cell migration from the medullary cavity to the repair area	[155]
	Helical microtransporters	MF	Provide suitable stem cell niche	Undegradable microrobot body	[161]
	Microrobots with NTS	MF	Enhanced cell adhesion	Undegradable microrobot body Unexplored cell-releasing	[164]
Osteosarcoma treatment	Hydrogel microrobots	MF	Controlled delivery of selective drugs	Unexplored microrobot delivery efficacy and therapeutic effect	[40]
	Spherical microscaffolds	MF	Initial development of microrobotic system for stem cell delivery to repair cartilage Targeted delivery of microrobot swarm	Unexplored microrobot delivery and therapeutic evaluation in vivo	[109]
Cartilage repair	Porous microrobots	MF	Complete microrobotic system for clinical application Exploration of stem cell delivery and its therapeutic effects in vivo	Targeting errors due to irregular injection rates	[106]
	Transformable microscaffolds	MF	Programmable shape and pore properties for treating specific diseases	Fail to provide a suitable microenvironment to better support various cell behaviors	[105]
	Porous microrobots	MF	No need of large space for magnetic actuation High fixation efficiency after targeted delivery in vivo	Non-minimal invasion Unknown biosafety of magnetic implants	[122]
	Achiral microswimmers	MF	Permitting long-distance targeted delivery Assembling into 3D structure for the chondrogenesis of stem cells	Biotoxic microrobot component (Ni)	[134]
	Janus microspheres	MF	Janus structure reduces cytotoxicity Natural and biocompatible microrobot material (alginate)	No in vivo experiments	[104]
	Spherical microscaffolds	MF	Developing wearable immobilization devices for microrobots	No evaluation of long-term fixation efficiency	[120]
	Size-controllable microscaffolds	MF	Sequential microrobot delivery to target cartilage and subchondral bone defects	Harmful degradation products	[125]
	Spherical microscaffolds	MF	Comprehensive evaluation of therapeutic effects of microrobots in vivo	No evaluation of targeted delivery of microrobots	[131]
RA treatment	Janus MnO_2_ nanomotors	CR	In vivo tracking of microrobots by ultrasound monitoring Removal of harmful substance	Unexplored degradability of nanomotors	[41]
	Janus Mg-based micromotors	CR	Ultrasound visualization Removal of harmful substance	Harmful degradation products	[107]
Muscle stimulation	Microswimmers responding to NIR	MF + light	Photothermal therapy for stimulating muscle contraction	Lack of in vivo tracking	[181]
Muscle regeneration	Helical micromotors	MF	Simulation of complex structure of muscle unit	No in vivo evaluation	[176]
Tendon repair	NO nanomotors with EXOs	CR	Enhanced tissue penetration of EXO Anti-inflammatory effects of reactive product (NO)	Lack of in vivo tracking	[108]

*EXOs* exosomes, *IONs* iron oxide nanoparticles, *MSCs* mesenchymal stem cells, *NIR* near-infrared, *NTS* nanostructured titanate surface, *PLGA* poly (lactic-co-glycolic acid), *RA* rheumatoid arthritis, *ROS* reactive oxygen species, *MF* magnetic field, *CR* chemical reaction, *NO* nitric oxide

#### Microrobots for Bone Regeneration

Bones are the support structures of human body and play a vital role in protecting internal organs from trauma, carrying much of the body's weight, and supporting movement in daily life. Hence, the most common bone-related disorders are fractures or bone defects that result in impaired daily function. In contrast to cartilage, injured bone tissue has a rich blood supply and consequently has a strong self-repairing capacity in response to mechanical stimuli [151]. However, the large bone defects caused by trauma, infection, and tumor resection, as well as the disorders of bone healing due to systemic diseases, like osteoporosis, remain a great challenge for orthopedic surgeons [152, 153]. Accordingly, there is still a need to develop new approaches to facilitate or accelerate the process of bone regeneration, especially in the context of systemic pathologies. In recent years, several researchers have attempted to develop microrobots for bone regeneration in response to the lack of reliable and responsive drug/cell transplantation systems. The matrix stiffness of bone tissue undergoes a dynamic change from soft to hard during bone growth or repair. Inspired by this dynamic process, Cao et al. fabricated biohybrid variable-stiffness soft actuators by combining the electroactive polymer Polypyrrole with alginate hydrogels [154]. Additionally, cell-derived plasma membrane nanofragments were immobilized in alginate hydrogels as bioactive components to induce rapid mineralization in the gels, thereby promoting bone formation [154]. Specifically, these actuators with variable stiffnesses were integrated into bone defects with the aid of an embedded device and attached to bone tissues through rapid mineralization. These biohybrid variable-stiffness actuators are compatible with the biology of bone formation and thus could be potential platform for bone repair. Besides the need to be in tune with the biology of bone formation, the primary use of microrobots applied in bone repair is to transport drugs/cells. In 2019, Singh et al. produced magnetically responsive hairbots by loading superparamagnetic iron oxide nanoparticles (SPIONs) into the cuticle and medulla of hair [155]. The reason for using hair as a skeleton for microrobots is that natural biomaterials derived from animals or humans have better biosafety and are more acceptable for current clinical applications [156]. Hair is a natural protein filament with physicochemical properties such as cysteine-rich, high elasticity, high mechanical strength, slow degradation, and good thermal insulation, and thus could be used for mass production of biocompatible microrobots [157]. In consequence, this microrobot whose raw material comes from the un-chemically treated human hair was named hairbot [155]. The hairbot had a thickness of about 10 µm and a lateral dimension of 60–80 µm and exhibited three modes of motion: rotation, rolling, and translational motion. In addition to the tiny size, the overlapping cuticle of hairbots presented a rough surface, which facilitated the adhesion of stem cells [158]. Furthermore, an external magnetic field was used to create a suitable rigid microenvironment around the hairbot to allow better differentiation of MSCs toward osteoblasts. It is important that the elasticity of hair mimics the stiffness suitable for osteoblast differentiation [159]. The medullary cavity of hairbots allowed it to perform more functions [160]. On the one hand, some osteogenesis-promoting drugs such as BMP-2 could be loaded into the medullary cavity, and on the other hand, the hollow medullary cavity of hairbots could serve as an excellent ultrasound contrast agent, especially in Doppler mode. The development of hairbots provided a microrobotic system that can be applied to bone repair, with the advantages of simple fabrication, low cost, and good response to magnetic fields.

In 2019, Yasa et al. designed a 3D-printed microrobotic cell transporter (MCT) to improve the efficiency of stem cell delivery and survivability, inspired by the flagella of bacteria [161]. The core material of the MCT is an inert branched derivative polymer of poly (ethylene glycol), trimethylolpropane ethoxylate triacrylate (TMPETA) containing SPIONs. Moreover, the MCT was designed as a double helix and has a nano-size with a length of about 76 μm and an inner cavity diameter of about 20 μm. Thanks to the double-helical structure, external magnetic fields could drive the MCT in a helical motion to ensure efficient propulsion in a liquid environment. Yasa et al. also found that the surrounding microenvironment of MCT could be modulated by biophysical and biochemical reprogramming, which enabled stem cells to leave the MCT and autonomously aggregate toward the injured tissues [162]. Analogous to the study of hairbots, Yasa et al. used two-photon lithography to regulate the mechanical properties of MCTs to create a rigid microenvironment for osteogenic differentiation [162]. Bone-enhancing drugs were also integrated into the MCT to synergistically promote osteogenic differentiation. Overall, the 3D-printed MCTs could intelligently respond to the external microenvironment and thereby enhanced the ability of stem cells to regenerate bone tissues.

In addition to regulating the fate of stem cells, reducing the loss of stem cells during transport is a practical problem that needs to be addressed. Specifically, the transport of microrobots in the human body is affected by body fluids, and as a result, the cells adhered on microrobots might be dislodged by fluids [163]. In 2021, Li et al. utilized 3D laser lithography to fabricate a magnetic microrobot with a bioactive nanostructured titanate surface (NTS) to modulate cell adhesion through substrate nanotopography [164]. The microrobot presented a burr-like porous spherical structure that enhanced magnetic responsiveness and cell-carrying capacity [163]. From a perspective of material fabrication, the chemical reaction between NaOH solution and nickel/titanium in the outer layer of the NTS generated the Na_2_Ti_2_O_4_(OH)_2_. The nanofiber modification by the titanate and hydroxyl group of Na_2_Ti_2_O_4_(OH)_2_ changed the surface of the microrobot from hydrophobic to hydrophilic, which was recognized to improve cell adhesion [165]. The results demonstrated that most of the MSCs remained tightly attached to the microrobots with NTS after fluid flushing at different volume flow rates. Biochemical results indicated that MSCs exhibited better cell viability and osteogenic differentiation in microrobots with NTS. In conclusion, nano-modification of microrobots improved the efficiency of cell transport, providing a new strategy to optimize the properties of cell-delivered microrobots.

In addition to cell-delivered microrobots, there is another type of microrobot that can respond dynamically to environmental changes by exploiting the rich chemical functionality of RNA molecules [166]. Inspired by natural bone nanostructures, Shen et al. developed an RNA-amorphous calcium phosphate nanomachine that could dynamically and programmatically induce the biomineralization of collagen scaffolds to simulate osteogenesis [167]. Self-assembled RNA was predominantly located in the center of the spherical nanomachine, while minerals were distributed in the outer layer. In this RNA nanomachine, non-covalent hydrogen bonding provided the energy source that initiates self-assembly of RNA molecules [168]. If there is a need to stop excessive osteogenesis, mineralization could be stopped by externally adding RNA-degrading enzymes to the RNA-biomineral nanomachine [169]. Since the RNA-biomineral nanomachines generated a microenvironment conducive to rapid collagen mineralization, the attachment, proliferation, and osteogenic differentiation of MSCs were significantly enhanced. The results showed that implantation of RNA-biomineral nanomachines induced more woven bone generation and these new bones were firmly attached to the mineralized scaffolds at the defect site in a mouse cranial bone defect model. Altogether, RNA-biomineral nanomachines mimicked the biological processes of natural bone formation and induced rapid and time-controlled intrafibrillar collagen mineralization. Exploring the potential of RNA in building functional nanomachines was important for bone regeneration engineering. In the future, RNA molecules could regulate the concentration of Ca^2^^+^ and be integrated into microrobotic delivery systems to induce bone mineralization.

Targeted drug delivery for treating osteosarcoma is another function of microrobots apart from bone repair [40]. In 2022, Mu et al. developed a magnetic field-controlled hydrogel microrobotic drug delivery system that allows for the precise delivery of drugs to kill tumors while reducing the side effects of drugs [40, 170]. This study used microrobots carrying EPZ015666, a PRMT5 inhibitor, to kill osteosarcoma. It has been shown that the speed of microrobots can be varied by changing magnetic field strength and frequency and can reach up to 100 μm s^−1^. Besides, microrobots could overcome blood flow resistance and be driven to the target site in vitro. However, whether microrobots can move in solid sarcomas remains to be investigated.

In summary, several microrobots were been developed to deliver stem cells and stimulate bone formation for bone repair and regeneration (Table 1), and exhibited several benefits: (1) better cellular transport capacity, including improved cell viability and enhanced cell adhesion; (2) the dynamic responses of microrobots to external physical and chemical signals can modulate local cell microenvironment to promote osteogenesis.

#### Microrobots for Skeletal Muscle and Tendon

Skeletal muscle accounts for approximately 40% of body mass and plays a critical role in metabolism and motor function [171]. As mentioned above, skeletal muscle repair requires a focus on muscle regeneration, angiogenesis, neurostimulation, and immunomodulation [172]. Muscle tissue engineering is one of the most promising therapeutic strategies for the future treatment of muscle injuries [173]. However, most of the current artificial muscle units were too simple in structure, which is still far from the ideal functional muscle tissue [174]. A multifunctional 3D microrobot integrating stem cells, biological factors, and physical stimulation could be another option for generating functionalized muscles [175]. In 2022, a study by Zhuge et al. proposed a muscle cell-loaded helical micromotor to generate the complex structures of muscle tissues. Microfluidics was used to encapsulate muscle cells along with magnetic IONs in a helical micromotor [176]. In this complex micromotor, the speed of movement is mainly varied by changing the helical pitch of microfibers, the concentration of IONs, and the strength of magnetic field. Benefiting from controlled helical motion, cellular micromotors could be assembled to form complex muscle units in a relatively safe and convenient manner [177]. Taken together, this study utilized the concept of microrobots to establish a bionic platform for muscle tissue repair, which provided a new idea for muscle tissue engineering.

In clinical practice, skeletal muscle stimulation is an effective approach to promote tissue repair and improve motor function [178]. Electrical stimulation was now widely used for muscle repair, but unstable electric fields might be harmful to skeletal muscle [179]. Therefore, optogenetics has been used as an alternative noninvasive treatment to improve the contractility of myotubes [180]. In 2022, Liu et al. designed a magnetically driven biohybrid microswimmer incorporated with near-infrared (NIR) stimulation [181]. The body of microswimmer was a naturally widespread subspecies of microalgae with good biocompatibility [182]. In addition, Fe_3_O_4_ nanoparticles with superparamagnetic properties and photothermal conversion capabilities enabled the microswimmer to respond to magnetic fields and NIR [183]. Driven by an external magnetic field, the microswimmers were driven to the exact muscle fiber that needed to be stimulated [184]. In addition, the microswimmers exhibited a highly stable photothermal conversion ability, and the temperature of the microswimmer rapidly increased by 5 °C after NIR irradiation, which effectively promoted muscle contraction. The specific mechanism probably was the interaction between actin and myosin induced by the elevated temperature [185]. In general, this study presented a drug/cell-free and efficient microrobot-based stimulation system, which provided a new treatment strategy for precise local muscle stimulation via the combination of magnetic actuation and photothermal stimulation.

In addition to skeletal muscles, tendons are also essential for body movement [57, 58]. Achilles tendinopathy is a common pathological condition, and current conservative and surgical treatments are less effective [186, 187]. Microneedle is a potential drug delivery system to treat Achilles tendinopathy, but mass residual drugs on the epidermal and dermal surfaces would reduce the therapeutic efficiency [188]. To address this problem, Liu et al. invented a microneedle array loaded with chemically driven nanomotors to transport EXOs [108]. In the injured tendons, the post-traumatic stress and inflammatory response induced a large amount of endogenous ROS production, which could be utilized by nanomotors to generate power through chemical reactions [25]. After transdermal administration via microneedle, L-arginine on the surface of nanomotors chemically reacted with ROS to produce nitric oxide, and the resulting driving force would actuate the EXOs to the deeper regions of tendon lesions [189]. The delivered EXOs eventually attenuated the inflammatory responses and promoted the proliferation and differentiation of tendon cells for tendon repair [190]. In this study, the combination of microneedles, nanomotors, and EXOs into a self-driven microrobotic system greatly improves the efficiency of EXOs to treat Achilles tendinopathy.

In muscle and tendon repair, these studies mentioned above have focused on stimulating tissue stimulation, functional unit reconstruction, and inflammation suppression rather than targeted delivery through microrobots (Table 1). Overall, there has been relatively little research on microrobots applied to skeletal muscle and tendon repair, possibly due to the lack of channels and cavities around muscles or tendons that would allow microrobot movement. However, microrobots could be used in tendon injuries of the shoulder and knee, such as rotator cuff and cruciate ligament repairs for targeted delivery.

## Microrobotic Systems-Based Delivery of Cells/Drugs

As discussed above, the integrated microrobotic delivery has brought a boost to the development of regenerative medicine in the musculoskeletal system. In addition to the design of microrobots, the precise actuation and real-time imaging in the microrobotic system-based delivery are also critical and will be discussed in detail.

### Actuation and Control of Microrobots in the Musculoskeletal System

Actuation technology is crucial for the motion of microrobots. Modes of microrobot motion are classified into two types depending on the power source, including self-driven and external power-driven. Self-driven motion generally refers to chemical propulsion including bubble generation [191], self-diffusiophoresis [192], self-electrophoresis [193], and the Marangoni effect propulsion [194], whereas external-driven one including magnetic, acoustic, and optoelectronic actuation [29–34]. The motion of microrobots can be designed according to the actual requirements for the treatments of different diseases.

**Fig. 5:**
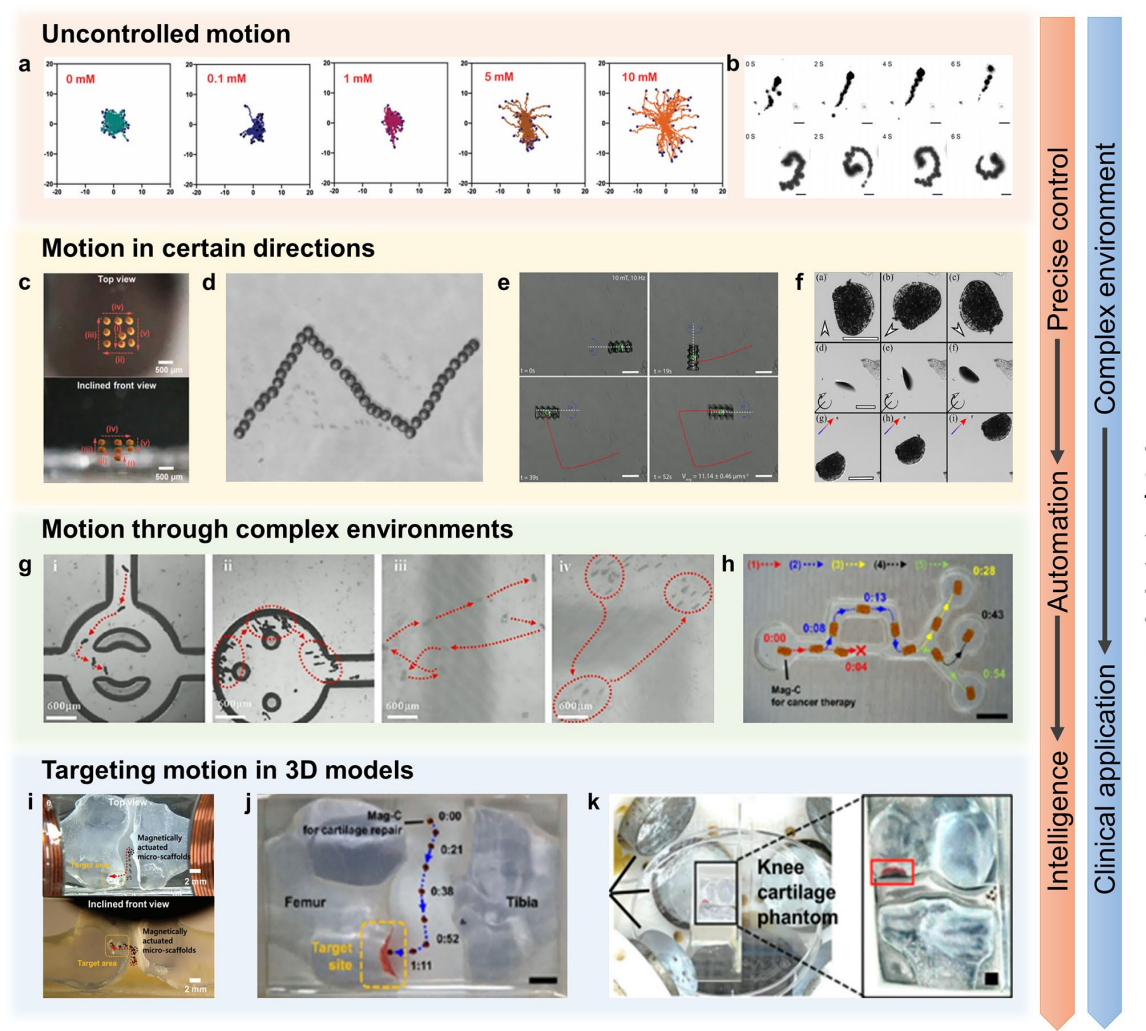
Different motion modes of microrobots. **a** Concentration-dependent autonomous diffusion [41]. Copyright (2022) John Wiley and Sons. **b** Linear and helical motions driven by chemical reactions [107]. Copyright (2021) American Chemical Society. **c** Linear motion in a gradient magnetic field [109]. Copyright (2017) John Wiley and Sons. **d** Trajectory of a microswimmer following a predesigned track of “N” [181]. Copyright (2022) American Chemical Society. **e** Spiral propulsion of helical microrobots under rotating magnetic fields [161]. Copyright (2019) John Wiley and Sons. **f** Spinning, rolling, and translation of hairbots [155]. Copyright (2019) Elsevier. **g** Move through complex channels by a combination of rolling and swimming motions [134]. Copyright (2023) American Chemical Society. **h** Targeted delivery through narrow channels [105]. Copyright (2020) American Chemical Society. **i**–**k** Targeted delivery in 3D knee models [105, 109, 125]. Copyright (2017) John Wiley and Sons, (2020) American Chemical Society, (2023) MDPI

Figure 5 illustrates the evolution of the motion of microrobots in the musculoskeletal system, including uncontrolled motion, motion in certain directions, motion in complex environments, and targeting motion in 3D models. In general, chemically driven microrobots usually exhibit uncontrolled motion modes (Fig. 5a, b). This is because these microrobots are typically used to remove harmful substances from the microenvironment of damaged tissues and move deeper into the lesions, driven by asymmetric chemical reactions. The MnO_2_ micromotor with a typical Janus structure designed by Xu et al. was self-driven by consuming excess H_2_O_2_ and generating O_2_ [195]. As the concentration of H_2_O_2_ increased, the motion pattern of micromotors changed from typical Brownian motion to outward diffusion. The results showed that the speed of the nanomotor reached 4.3 μm s^−1^ in 10 mM H_2_O_2_ solution, which was 2.7 times higher than that without H_2_O_2_. In simulated synovial fluid (SSF) with 1 mM H_2_O_2_ (with higher viscosity), the nanomotor could reach a speed of 3.4 μm s^−1^. The outward diffusion of micromotors in H_2_O_2_-abundant microenvironment significantly improved the diffusion efficiency of the generated O_2_ in the knee joint for RA treatment [196]. Similarly, in the study of Xu et al., the asymmetric Mg-based micromotor generated hydrogen by interacting with ROS in the surrounding environment and propelled itself in a linear or helical motion (Fig. 5b) [107]. When Mg was depleted, the micromotor gradually stopped moving, and the average time of movement was about 5 min. In SSF and PBS, the speeds of Mg-HA motors were 40.1 and 45.5 μm s^−1^, respectively; moreover, the average diffusion length (10 s) was about 220.84 and 266.7 μm in the two liquids, respectively. This motion greatly improved the diffusion efficiency of hydrogen in the joint cavity to remove excess inflammatory factors.

Unlike self-driven actuation, external power-driven actuation is typically controllable and is often used for the targeted delivery of drugs/cells. In the musculoskeletal system, the injury region can be clearly detected, so the high accuracy of magnetic actuation in 3D would maximize the efficiency of targeted delivery. To achieve the precise magnetic actuation, the influence of fluid resistance on microrobot motion should be considered after in vivo transplantation. In order to explore the feasibility of magnetic actuation in the musculoskeletal systems, the researchers first explored the movement of microrobots along a specific direction in a fluidic environment. In 2018, Go et al. designed a magnetic device consisting of eight electromagnetic coils and a pure iron core. The electromagnetic coils were capable of generating uniform and gradient magnetic fields of up to about 45 mT and 1.8 T m^−1^ and ultimately enabling the motion of microrobots in five degrees of freedom [109]. It was found that the velocities of microscaffolds in the *x*- and *z*- axes showed a linear increase with the raised gradient magnetic field. In glycerin solution (70% (v/v)), the velocities of microscaffolds were about 100 and 200 μm s^−1^ when the gradient magnetic field is 0.9 and 1.8 T m^−1^, respectively. It is worth noting that the velocities along the *x*-axis are slightly higher than those along the *z*-axis, which may be due to the effect of gravity.

The motion of a single microrobot in a particular direction in 3D space can be described by the following model (Fig. 5c):

1 $$F_{{{\text{mag}}}} - F_{{{\text{drag}}}} - F_{{{\text{gravity}}}} = \, 0$$

*F*_mag_, *F*_drag_, and *F*_gravity_ are the magnetic, fluid resistance, and gravity forces that act on the microrobot, respectively [55]. The fluid resistance can be expressed as:

2 $$F_{{{\text{drag}}}} = \, 6\pi \mu RV$$

*R*, *V*, and *µ* are the radius, moving speed, and viscosity coefficient of the microrobot, respectively. Thus, with an increase in fluid viscosity, the moving speed of the microrobot decreased, accordingly. In addition to movement within joints, the microrobots could also move in specific directions in bone and muscle tissues. Since there is not enough space around bone and muscle tissues, microrobots generally roll on the tissues [197]. Liu et al. used a homemade triaxial Helmholtz coil to generate a uniform rotating magnetic field to drive the microswimmers toward the desired region (Fig. 5d) [181]. In a homogeneous rotating magnetic field of 7 mT, the rotation of the microswimmer was transformed into translation on the muscle surface by friction. The velocity of the microswimmer was related to the rotating frequency, and the motion of the microswimmer reached a peak velocity of 17 μm s^−1^ when the frequency was 8 Hz in DI water. This indicated that 8 Hz was the step-out frequency of the microswimmer, while above it the viscous resistance would exceed the maximum magnetic torque. Likewise, inspired by flagellar motion, the helical MCTs designed by Yasa et al. were driven by a rotating magnetic field [161]. The torque applied to the helical MCT by the rotating magnetic field actuated it to rotate, which enabled it to move longitudinally in a low Reynolds number fluid environment (Fig. 5e) [198]. It is remarkable that the torque of the cell-free MCT in a rotating magnetic field of 10 mT and 10 Hz overcame the resistive force, drag and surface friction, and enabled the MCT to swim at a speed of 11.14 μm s^−1^. In the study of hairbots, magnetic actuation was mainly manifested in three different modes: rotational motion, rolling motion, and transverse motion [155]. A rotating magnetic field applied in-plane allowed the hairbot to rotate freely on the surface (10 mT, 10 Hz); a rotating magnetic field applied out-of-plane allowed the hairbot to roll vertically on the surface (10 mT, 10 Hz), and a gradient magnetic field applied in-plane allowed the hairbot to perform a translational motion (1 mT, 2 T m^−1^) (Fig. 5f).

Furthermore, researchers have begun to explore the motion of microrobots in complex environments. The motion of waffle-shaped microswimmers was mainly controlled by a rotating magnetic field generated by a 3D Helmholtz coil system [134]. In this study, the motion pattern, speed, and direction of a set of microswimmers could be manipulated by varying parameters such as magnetic field strength, rotation frequency, and rotation plane angle. The maximum swimming speed of the microswimmer was about 60–70 μm s^−1^ (5 mT, 6.5 Hz) in a homogeneous rotating magnetic field in liquids with different viscosities. Interestingly, the microswimmer consistently rolled faster than it swam at the same frequency, with a maximum rolling speed of about 250–300 μm s^−1^ (5 mT, 5 Hz). This further indicated that it is possible to drive the microswimmer through different terrains by changing the frequency (Fig. 5g). Commendably, Go et al. conducted a series of studies using magnetically driven microrobots to treat cartilage injuries and have developed their system [105]. In 2020, given that three translational degrees of freedom were required for movement within the joint, they used a circular microrobot with a minimal velocity difference (body ratio: 1) for motion testing in a complex model (Fig. 5h). In PBS, the velocity of the spherical microrobot increased linearly with the increase in external magnetic field and gradient. The maximum velocity of the microrobot was about 2.8 mm s^−1^ (40 mT and 1 T m^−1^). This velocity of motion was undoubtedly well above the velocities of the micro/nanorobots in all previous studies. For further simulating magnetic actuation within the knee joint, 3D models and in vivo models were used to validate the feasibility of magnetic actuation (Fig. 5i–k). In 2020, the EMA system developed by Go et al. could generate magnetic fields and gradients of up to 80 mT and 1.2 T m^−1^ in the 20 mm range [106]. The velocity of the microrobot increased linearly with the gradient magnetic field and could reach a maximum velocity of 3.6 mm s^−1^ (40 mT, 0.6 T m^−1^) in PBS. In this system, the average velocity of the microrobots loaded with cells was 2.88 mm s^−1^ (30 mT, 0.6 T m^−1^), which was slightly lower than that of the microrobots without cells. A soft magnetic core was inserted in the center of the coil to generate a stronger magnetic field. For precise control within the knee joint, the current (***i***) used on the plurality of electromagnetic coils of the EMA system was calculated by a pseudo-inverse on account of the desired magnetic field (***B***) and magnetic force (***F***) and can be represented as:

3 $${i} = {\mathcal{A}}_{B,F} \left( {M,P} \right)^{\dag } \left[ {\begin{array}{*{20}c} {\varvec{B}} \\ {\varvec{F}} \\ \end{array} } \right] = \left[ {\begin{array}{*{20}l} {\begin{array}{*{20}c} {\begin{array}{*{20}c} {{\varvec{B}}\left( P \right)} \\ {{\varvec{M}}^{T} \frac{{\partial {\varvec{B}}\left( P \right)}}{\partial x}} \\ \end{array} } \\ {{\varvec{M}}^{T} \frac{{\partial {\varvec{B}}\left( P \right)}}{\partial y}} \\ \end{array} } \hfill \\ {{\varvec{M}}^{T} \frac{{\partial {\varvec{B}}\left( P \right)}}{\partial z}} \hfill \\ \end{array} } \right]^{\dag } \left[ {\begin{array}{*{20}c} {\varvec{B}} \\ {\varvec{F}} \\ \end{array} } \right]$$

The 3 × *n* matrices ${\varvec{B}}(P)$, $\frac{\partial {\varvec{B}}(P)}{\partial x}$, $\frac{\partial {\varvec{B}}(P)}{\partial y}$, $\frac{\partial {\varvec{B}}(P)}{\partial z}$ were the magnetic fields and gradient produced in *n* coils per unit of current at the point (*P*) of the workspace. (*M*, *P*) ∈ R6 × *n* was the drive matrix of the EMA system. The pseudo-inverse of (*M*, *P*) was decomposed using the singular value decomposition  = ***U***∑^*T*^. ***U*** was the *n* $\times$ *n* orthogonal matrix whose columns were ^*T*^; was the *n* $\times$ *n* orthogonal matrix with columns ^*T*^ singular vectors and ∑ is a 6 × *n* diagonal matrix whose diagonal terms $\sigma$_*i*_ are the singular values of ${\mathcal{A}}$. In addition to the use of the EMA system, the team from South Korea has innovatively proposed a wearable device that consists of a magnet array device and a paramagnetic implant [122]. In this study, the paramagnetic implant was permanently embedded in the subchondral bone at the defect. The magnetic array device around the knee joint would magnetize the paramagnetic implant and attract microrobots to the cartilage defect. This was a very interesting innovation that proposes a targeting and fixation system to overcome the limitations of EMA system, such as no fixation of microrobots after targeting, lack of working place, high cost, and low stability. Through the synergistic effect of the magnetic array device and the paramagnetic implant, the microrobot was attracted to the injury site by a strong magnetic field. Conceivably, unlike the EMA system, the velocity of microrobots increased exponentially as the distance was shortened. The results showed that the targeting efficiency of the system exceeded 90%.

To summarize, the actuation and control systems of microrobots in the musculoskeletal systems are designed according to the different therapeutic purpose. With the optimization of actuation devices and control mechanisms, microrobots can move in different modes in complex environments to complete targeted delivery or therapeutic tasks precisely and intelligently after in vivo transplantation. In different application environments, microrobots exhibit distinct motion characteristics, which are summarized in Table 2.

**Table 2:** Summary of actuation characteristics of microrobots in the musculoskeletal system

Actuation types	Types of microrobots	Power source (device)	Application environments	Actuation speed (maximum)	Motion modes	References
Magnetic actuation	Microscaffolds	Gradient magnetic fields (EMA system)	Glycerin solution (70% (v/v))	200 μm s^−1^ (45 mT, 1.8 T m^−1^)	Linear movement	[109]
	Janus microspheres	Gradient magnetic fields (EMA device)	N/A	N/A	Linear movement Rotation	[104]
	Porous microrobots	Gradient magnetic fields (EMA system)	PBS	3.6 mm s^−1^ (40 mT, 0.6 T m^−1^)	Linear movement	[106]
			Glycerin solution (70% (v/v))	150 μm s^−1^ (30 mT, 0.6 T m^−1^)		
	Spherical microrobots	Gradient magnetic fields (EMA system)	PBS	2.8 mm s^−1^ (40 mT and 1 T m^−1^)	Linear movement	[105]
			10% FBS	1.75 mm s^−1^ (ditto)		
			0.1% HA	100 μm s^−1^ (ditto)		
	Porous microrobots	Strong magnetic attraction (magnetic implant system)	N/A	N/A	Linear movement (passive movement)	[122]
	Hairbots	Magnetic gradient and rotating fields	N/A	N/A	Linear movement, rotation, rolling	[155]
	Helical MCT	Rotating magnetic fields	Newtonian fluids	11.14 μm s^−1^ (10 mT, 10 Hz)	Rotation → corkscrew motion	[161]
	Achiral 2D microswimmers	Rotating magnetic fields (3D Helmholtz Coil System)	PBS/ACF/MS	60–70 μm s^−1^ (5 mT, 6.5 Hz)	Swim	[134]
				250–300 μm s^−1^ (ditto)	Rolling	
	Helical micromotors	Rotating magnetic fields	5 wt% PVA	0.06 mm s^−1^	Rotation → corkscrew motion	
			1 wt% CaCl_2_	0.3 mm s^−1^		[176]
	Spherical microswimmers	Rotating magnetic fields (3D Helmholtz Coil)	DI water	17 μm s^−1^ (7 mT, 8 Hz)	Rotation → linear translation	[181]
			Diluted blood	6 μm s^−1^ (ditto)		
			CGM/plasma	8–10 μm s^−1^ (ditto)		
Chemical reaction	Janus MnO_2_-motors	H_2_O_2_ decomposition and O_2_ generation	H_2_O_2_ solutions	4.3 μm s^−1^	Diffusion	[41]
			SSF	3.4 μm s^−1^		
	Janus Mg − HA motors	ROS decomposition and H_2_ generation	PBS	45.5 μm s^−1^	Diffusion	[107]
			SSF	40.1 μm s^−1^		

*EMA* electromagnetic actuation, *N/A* not applicable, *PBS* phosphate buffered solution, *FBS* fetal bovine serum, *HA* hyaluronic acid, *MCT* microrobotic cell transporter, *ACF* artificial cerebrospinal fluid, *MS* mouse serum, *PVA* polyvinyl alcohol, *DI* deionized, *CGM* cell growth media, *SSF* simulated synovial fluid, *ROS* reactive oxygen species

### Imaging-Guided Microrobotic Delivery System

The imaging system is complementary and closely linked to the actuation system [163]. Most of the current microrobot research in the musculoskeletal system remained in the in vitro experimental stage [161]. As a result, most studies have been devoted to the fabrication of microrobots or the upgrading of manipulation systems without considering in vivo imaging [199]. Due to the minimally invasive targeting properties of microrobots, there is a greater need for well-designed imaging systems to guide their movements in real-time [200].

**Fig. 6:**
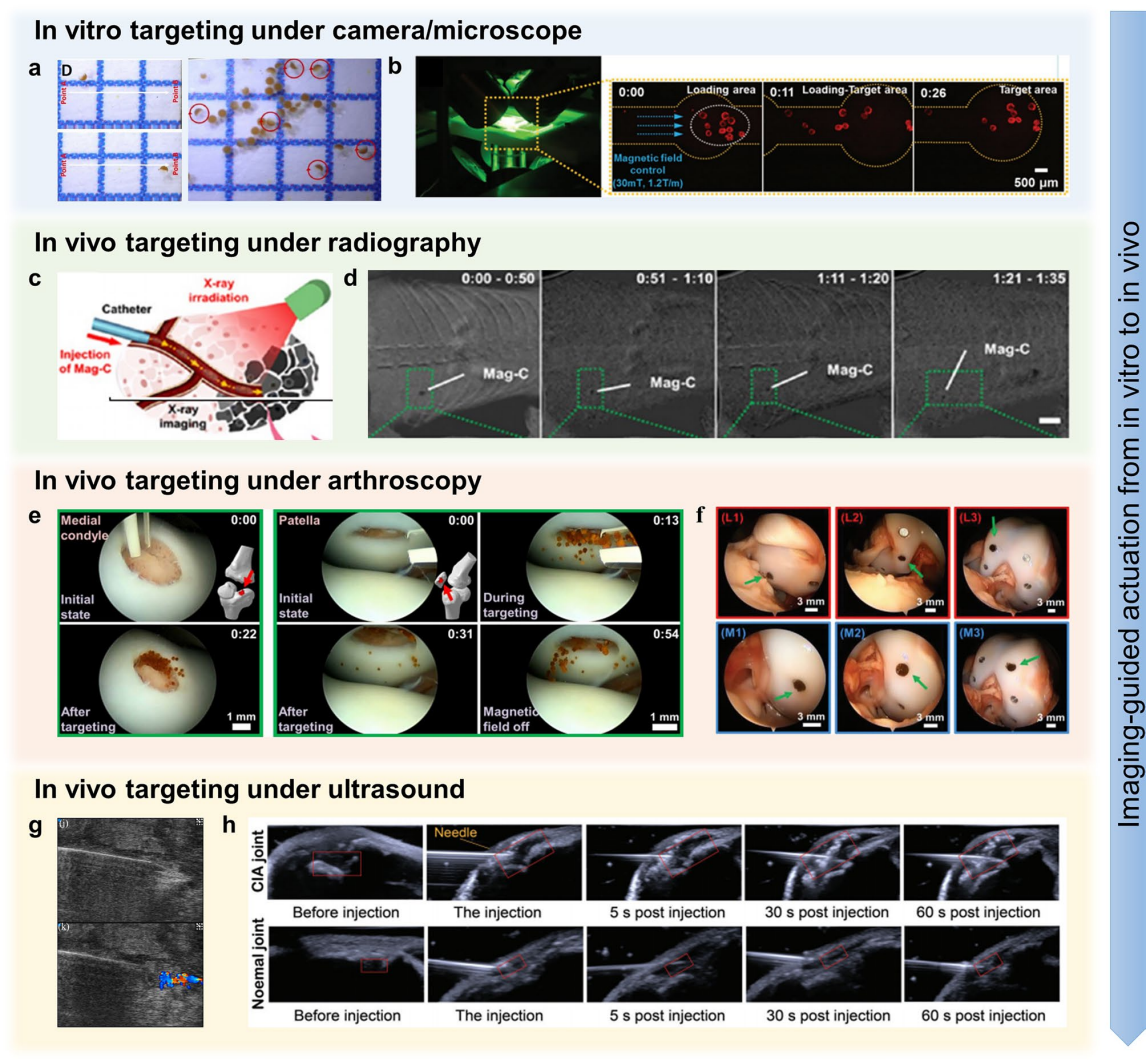
Imaging-guided delivery of microrobots from in vitro to in vivo. **a** Microsphere motions recorded by the camera [104]. Copyright (2018) Elsevier. **b** Microrobot motions recorded by fluorescence microscopy [109]. Copyright (2017) John Wiley and Sons. **c** Schematic of X-ray guided microrobot delivery [105]. Copyright (2020) American Chemical Society. **d** Imaging of microrobots motion using X-ray imaging in the thoracic cavity of rats [105]. Copyright (2020) American Chemical Society. **e** Targeted delivery under arthroscopy [106]. Copyright (2020) American Association for the Advancement of Science. **f** Magnetic implant targeting system under arthroscopy [122]. Copyright (2021) John Wiley and Sons. **g** Hairbots imaging under ultrasound [155]. Copyright (2019) Elsevier. **h** Ultrasound imaging of the diffusion of self-driven nanomotors [41]. Copyright (2022) John Wiley and Sons

Figure 6 illustrates the gradual refinement of imaging-guided delivery systems from in vitro to in vivo in the musculoskeletal system. Initially, the motion of microrobots in 3D Helmholtz coils could only be viewed with a camera or microscope (Fig. 6a, b). Clearly, such in vitro imaging is still far from the practical application of microrobots. For in vivo applications, the real-time imaging for precise and adaptive microrobot delivery should be considered into the design of microrobotic systems. In 2021, an iodine-based contrast agent was loaded into the microrobot and two orthogonal X-ray devices were used for in vivo imaging. In the rat thoracic cavity, the image of the microrobot gradually changed from clear to blurred and finally disappeared as the contrast agent diffused out of the gelatin (Fig. 6c, d). In an era of growing enthusiasm for minimally invasive treatments, many knee disorders can be treated using arthroscopy. Most studies by Go et al. have used knee arthroscopy to visualize the movement of microrobots in vivo (Fig. 6e, f) [201]. Arthroscopy allows direct visual observation of various structures in the knee joint cavity, helping the operator to fully understand and assess the lesions, with the advantages of high definition, high accuracy, and excellent real-time performance. However, although the delivery of microrobots is performed by needle injection, knee arthroscopy itself is an invasive treatment, which might increase the risk of incision infection and venous thrombosis [202, 203]. Besides, arthroscopy is not capable of presenting the deep cartilage lesions and subchondral bone alterations [204].

Notably, ultrasound, as a noninvasive, radiation-free examination, has also been used to observe and guide the movement of microrobots in the musculoskeletal system (Fig. 6g, h) [45]. In the study of hairbots, they could be well captured by ultrasound because they had a hollow medullary cavity. Subsequently, in color Doppler mode, the color of the hairbot depended on the speed of its movement [205]. In self-driven microrobots, bubbles generated by chemical reactions were visualized by ultrasound to track the active motion of microrobots in real-time. Moreover, the intensity of the echo signal reflected the local oxygen production, which also helped to accurately assess the degree of intra-articular inflammation. Therefore, ultrasound is increasingly used as an inexpensive, noninvasive, and easy-to-use tools in microrobotic imaging systems [112].

## Summary and Outlook

Musculoskeletal system and regenerative medicine are the field with significant prospects in modern medicine. In the last decade, with the continuous development of nanofabrication and control technologies, microrobots have shown unrivaled promise for biomedical applications. This review of microrobots applied in the musculoskeletal system focuses on microrobot design, drive mechanisms and devices, imaging system for tracking, and in vivo applications. Unlike traditional therapeutic methods and tissue engineering, microrobotic systems are characterized by precise targeting, real-time imaging, and remote regulation.

Although the individual tissues of the musculoskeletal system vary in morphology, structure, and function, the same goal of microrobots applied to bone, cartilage, muscle, and tendon is to repair the injured tissues. Table 3 summarizes the critical factors and challenges that need consideration for microrobots applied in the musculoskeletal system.

**Table 3:** Critical factors and challenges to apply microrobots in the musculoskeletal system

Objectives	Critical factors and challenges	Typical examples of achievements	References
Bone	Suitable microrobot properties to support the behaviors and functions of delivered stem cells	External magnetic fields enhance the osteogenic differentiation of MSCs through enhanced calcium response	[154]
	Strong bioactivities to induce rapid bone formation in situ	Bioinduced variable-stiffness devices adhere and integrate onto bone tissue after mineralization RNA-ACP nanomachine simulates osteogenesis in a dynamic and programmable manner	[155, 167]
Cartilage	Biocompatible core materials of microrobots	Janus microsphere segregates IONs from MSCs completely FDA-approved biomaterials: chitosan-based scaffolds	[107]
	Microrobot immobilization after targeted delivery	Wearable magnets based on the Halbach array principle immobilized the microrobots at the target site	[120]
RA	In situ regulation of pathological microenvironment	Nanomotors consume hazardous substances and generate beneficial substances	[41, 107]
Muscle	Engineering complex muscle tissue units	Helical cell micromotors assemble to mimic muscle units	[176]
	Noninvasive system to stimulate skeletal muscle	Wireless and precise muscle stimulation by using magnetic microswimmers in combination with NIR	[181]

*ACP* amorphous calcium phosphate, *FDA* Food and Drug Administration, *IONs* iron oxide nanoparticles, *MSCs* mesenchymal stem cells, *RA* rheumatoid arthritis, *NIR* near-infrared

In the musculoskeletal system, the main function of microrobots is to deliver drugs/cells, so their design ought to fulfill several requirements. First, as a "Noah's Ark" with a drive system, both the "ocean" (human body) and the "passengers" (cells) require the good biocompatibility and low toxicity of microrobots [206]. As a result, many studies have opted for more biocompatible natural biomaterials (chitosan and sodium alginate) to fabricate microrobots [104, 105, 122]. The degradation products of microrobots should have little or no effect on the microenvironment of the treated tissues. In addition, some special designs such as Janus structure that avoid direct contact between cells and magnetic particles are proposed. Finally, for magnetic field-driven microrobots, the concentration of magnetically responsive substances is expected to meet the magnetic drive requirements without causing cytotoxicity.

Second, actuation technology is clearly at the heart of microrobotic systems to improve target delivery efficiency. In the musculoskeletal system, the mechanisms that drive the microrobots mainly include self-drive and external-drive. Generally, chemically driven microrobots generate the force of propulsion in a certain direction through their chemical reactions with the harmful substances in local tissue environment. The speed and diffusion of microrobots gradually increase with the concentration of the reactants. Self-driven microrobots energized by chemical reactions have limitations in motion control, making them suitable for diseases with low targeting requirements [41]. Of all the actuation methods, magnetic actuation is the most viable method for navigating microrobots in human body. In general, magnetic actuation is characterized by the precise control and targeted delivery. The driving power mainly consists of gradient magnetic fields and rotating magnetic fields. Gradient magnetic fields generally drive the microrobots to move linearly in a specific direction. However, the effect of rotating magnetic fields varies depending on the shape of microrobots. When the microrobots are spherical or achiral structures, the rotating magnetic fields would make them move in a rotating or rolling motion. When the microrobot is helical, the torque applied by the rotating magnetic fields pushes it forward in a helical motion. The original electromagnetic actuation device was three-axis Helmholtz coils where the motion of the microrobots could be observed under a microscope. With the gradual improvement of the control mechanism, electromagnetic systems were designed with more consideration for application scenarios. Some multi-axis coil systems were used for fine manipulation in larger spaces. Additionally, the magnetic field could trigger other functions of the microrobot, such as deformation, heating, and magneto-electricity. However, a major challenge for magnetic-driven microrobots is the choice of magnetic core material [24]. Iron oxide-based nanoparticle is a potential candidate with good biocompatibility and excellent responsiveness to magnetic fields [24, 106]. Besides, since the magnetic field decays rapidly with increasing spatial distance, a relatively large space and power supply are required to ensure the proper operation of the magnetic-driven system. With the development of magnetic-driven systems, the control of single or several microrobots has evolved into the control of the collective behavior of the microrobotic swarm [44, 45, 111]. In addition to magnetically driven microrobots, light-responsive microrobots have also shown great potential for applications in the musculoskeletal system. Since tendons and muscles are tissues located superficially in the body, external light irradiation could well penetrate the skin and guide the microrobots for photothermal treatment [207–209].

Third, considering the practical scenarios of microrobots, the tracking of microrobots after implantation is equally crucial. Imaging systems not only localize lesions and guide delivery through real-time imaging, but also monitor therapeutic efficacy after treatments. The development of microrobotic swarm technology made it possible to track the microrobots in micro- and even nanoscales. In complex biological environments, ultrasound Doppler imaging is an essential noninvasive means of swarm navigation. In our previous work, 3D blood flow induced by magnetically controlled microrobots could be captured by Doppler signals for real-time tracking. This imaging modality is radiation-free and portable and thereby easy to perform at the bedside [44, 112, 205]. Another promising modality for real-time imaging of micro/nanorobots is fluorescence imaging. Notably, fluorescent micro/nanorobots have the advantages of optical traceability, environmental responsiveness, and targeting photon-induced cytotoxicity [210].

**Fig. 7:**
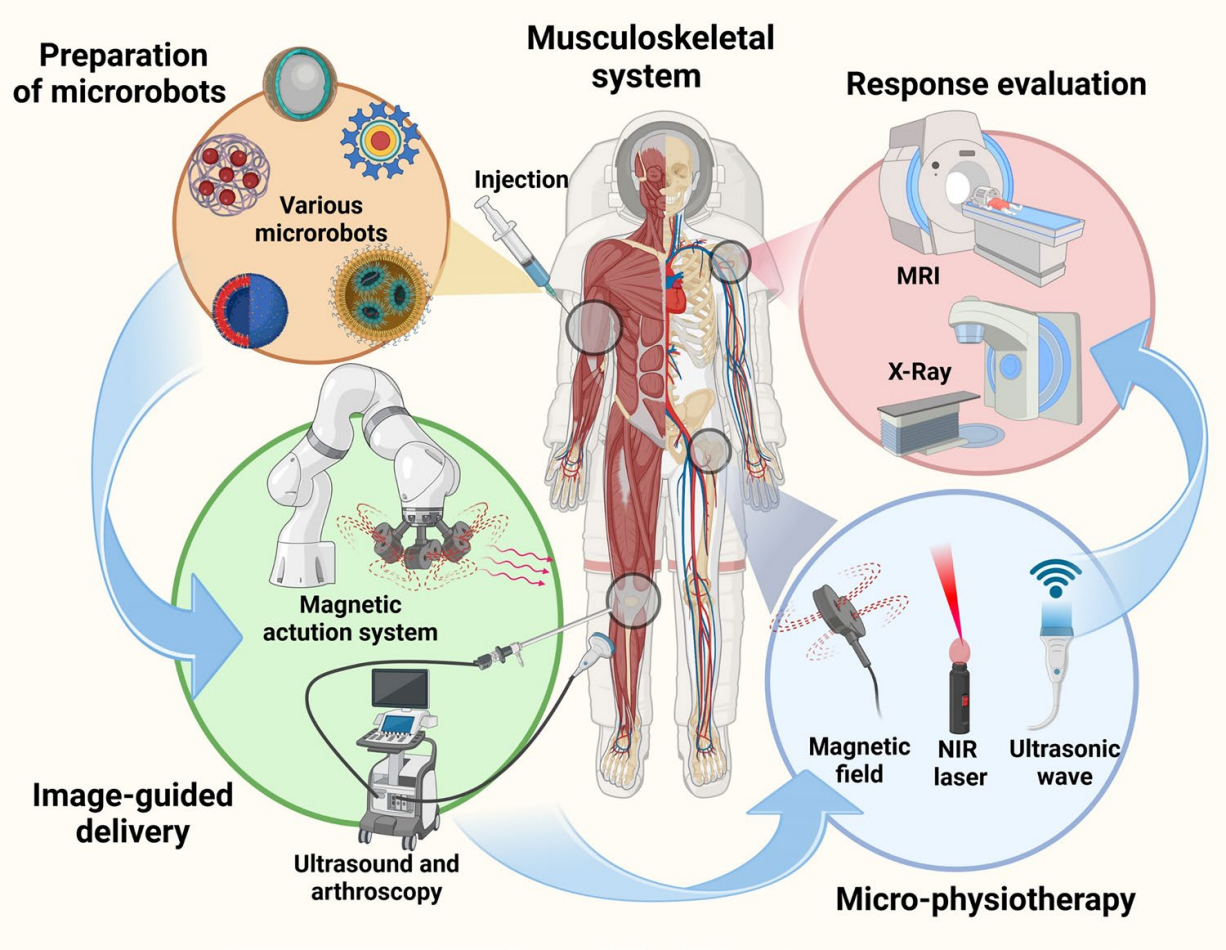
Prospects for clinical translation of microrobotic systems in the musculoskeletal system in the future

In addition to common orthopedic diseases, deep vein thrombosis of the lower extremities, as one of the most common complications of MSDs, also brings great trouble to surgeons. In terms of current practice, filters are used to prevent thrombus dislodgement. Notably, Guan et al. developed a heparinoid-polymer brush biointerfacing strategy for swarming magnetic nanorobots for in vivo thrombolysis. This remarkable targeted delivery platform provided a new strategy for the treatment of deep vein thrombosis [211]. In the future, microrobot research in the musculoskeletal system ought to focus on in vivo animal experiments, especially large animal experiments. Although microrobots have shown a great potential to improve the therapeutic effects of stem cell/drug delivery, as noted by Nordberg et al., understanding the barriers to clinical translation is critical to advancing microrobotic products [212]. As shown in Fig. 7, researchers should endeavor to drill down on the following key points of the microrobotic system for MSD treatment: (1) Use biocompatible and clinically safe materials approved by FDA. (2) Improve the efficiency of drug/cell targeting and immobilization through better control systems. (3) Imaging systems are needed to monitor the effects of drug delivery as well as to support post-delivery efficacy assessment. (4) Development of in situ modulation strategies after target immobilization, including magnetic intervention, synergistic therapy with bioactive factors, and photothermal therapy. (5) Invention of a clinically appropriate mobile multi-degree-of-freedom wireless actuation/control system.

Microrobots have gradually moved from a remote frontier technology to a clinical reality. We are supposed to focus on solving the current core problems by bringing together researchers in the fields of medicine, mechanical engineering, materials science, and biology to tackle the clinical translation of microrobotic systems in the musculoskeletal system and regenerative medicine.
